# Seasonal and interannual risks of dengue introduction from South-East Asia into China, 2005-2015

**DOI:** 10.1371/journal.pntd.0006743

**Published:** 2018-11-09

**Authors:** Shengjie Lai, Michael A. Johansson, Wenwu Yin, Nicola A. Wardrop, Willem G. van Panhuis, Amy Wesolowski, Moritz U. G. Kraemer, Isaac I. Bogoch, Dylain Kain, Aidan Findlater, Marc Choisy, Zhuojie Huang, Di Mu, Yu Li, Yangni He, Qiulan Chen, Juan Yang, Kamran Khan, Andrew J. Tatem, Hongjie Yu

**Affiliations:** 1 School of Public Health, Fudan University, Key Laboratory of Public Health Safety, Ministry of Education, Shanghai, China; 2 WorldPop, Department of Geography and Environment, University of Southampton, Southampton, United Kingdom; 3 Division of Infectious Disease, Key Laboratory of Surveillance and Early–warning on Infectious Disease, Chinese Center for Disease Control and Prevention, Changping District, Beijing, China; 4 Flowminder Foundation, Stockholm, Sweden; 5 Dengue Branch, Division of Vector-Borne Diseases, Centers for Disease Control and Prevention, San Juan, Puerto Rico; 6 Center for Communicable Disease Dynamics, Harvard T.H. Chan School of Public Health, Boston, MA, United States of America; 7 Department for International Development, London, United Kingdom; 8 Epidemiology and Biomedical Informatics, University of Pittsburgh, Pittsburgh, PA, United States of America; 9 Department of Epidemiology, Johns Hopkins Bloomberg School of Public Health, Baltimore, MD, United States of America; 10 Harvard Medical School, Harvard University, Boston, MA, United States of America; 11 Computational Epidemiology Lab, Boston Children’s Hospital, Boston, MA, United States of America; 12 Department of Zoology, University of Oxford, New Radcliffe House, Radcliffe Observatory Quarter, Oxford, United Kingdom; 13 Department of Medicine, Division of Infectious Diseases, University of Toronto, Toronto, ON, Canada; 14 Divisions of General Internal Medicine and Infectious Diseases, University Health Network, Toronto, ON, Canada; 15 Department of Medicine, University of Toronto, Toronto, ON, Canada; 16 MIVEGEC, IRD, CNRS, University of Montpellier, Montpellier, France; 17 Oxford University Clinical Research Unit, National Hospital for Tropical Diseases, Hanoi, Vietnam; 18 Li Ka Shing Knowledge Institute, St Michael’s Hospital, Toronto, Ontario, Canada; Institute for Disease Modeling, UNITED STATES

## Abstract

Due to worldwide increased human mobility, air-transportation data and mathematical models have been widely used to measure risks of global dispersal of pathogens. However, the seasonal and interannual risks of pathogens importation and onward transmission from endemic countries have rarely been quantified and validated. We constructed a modelling framework, integrating air travel, epidemiological, demographical, entomological and meteorological data, to measure the seasonal probability of dengue introduction from endemic countries. This framework has been applied retrospectively to elucidate spatiotemporal patterns and increasing seasonal risk of dengue importation from South-East Asia into China via air travel in multiple populations, Chinese travelers and local residents, over a decade of 2005–15. We found that the volume of airline travelers from South-East Asia into China has quadrupled from 2005 to 2015 with Chinese travelers increased rapidly. Following the growth of air traffic, the probability of dengue importation from South-East Asia into China has increased dramatically from 2005 to 2015. This study also revealed seasonal asymmetries of transmission routes: Sri Lanka and Maldives have emerged as origins; neglected cities at central and coastal China have been increasingly vulnerable to dengue importation and onward transmission. Compared to the monthly occurrence of dengue reported in China, our model performed robustly for importation and onward transmission risk estimates. The approach and evidence could facilitate to understand and mitigate the changing seasonal threat of arbovirus from endemic regions.

## Author summary

Given the global expanding distribution of *Aedes* mosquitoes, dengue has established itself throughout the world’s tropical and subtropical regions in both endemic and epidemic transmission cycles, causing significant morbidity and mortality. Moreover, the rise of air travel over the past century has resulted in a highly inter-connected world, where geographical distance is becoming less of a barrier to pathogen dispersal. However, few studies have quantified and validated changes in seasonal and long-term risks of international spread for infectious diseases. In China, dengue remains a seasonal disease occasionally triggered by imported dengue viruses in travellers, with more than 90% of imported cases between 2005 and 2014 originated from South-East Asia. Therefore, taking dengue importation from South-East Asia into China as an example, we constructed a branching process modelling framework to integrate three components for assessing the risk of dengue introduction into a country: 1) the risk of a person acquiring the disease in the origin country; 2) the probability of a person traveling to the destination country of interest while infectious; and 3) the likelihood of subsequent local transmission in the destination country. This model has revealed the seasonal patterns and increasing risks in routes of dengue spread by air travel over a decade. The spatiotemporal heterogeneities of dengue importation risk have also been seen in the travelers of Chinese and SEA residents. The risk of introduced transmission from particular routes highlighted could be used to inform efforts to dengue prevention and control, particularly in currently neglected, high-risk locations.

## Introduction

The substantial growth and reach of human travel in recent decades has contributed to the global spread of infectious diseases [1–4]. In particular, air travel has allowed human hosts or carriers of pathogens to move long distances within the incubation period of infections [5], such as the viruses that cause severe acute respiratory syndrome (SARS), H1N1, Ebola, Zika, and yellow fever [6–11], or the parasites that cause malaria [12–14]. Regarding to the continual growth of international tourist arrivals, from 25 million in 1950 to 1.2 billion in 2015 [15], understanding the global dynamics of infectious disease has become a major 21st-century challenge, and mechanistic or mathematical models built with air-transportation data been widely used to measure risks of arriving infected humans, growth rate of an introduced epidemic and the impact of specific surveillance and control strategies [2, 6, 16, 17].

Some relevant factors for assessing the risk of disease importation from endemic regions into a country are: 1) the risk of a person acquiring the disease in the origin country; 2) the risk of a person traveling to the destination country of interest while infectious; and 3) the likelihood of subsequent local transmission in the destination country [18]. However, most previous modelling studies have only focused on some of these components, and the seasonal and inter-annual risks of international spread of infectious diseases have rarely been quantified [6, 16, 19–22]. Moreover, the relative exposure risk and importation probability in travelers are likely to differ between local residents in endemic regions and residents of non-endemic areas traveling to endemic countries [21, 23, 24].

Given the global expanding distribution of *Aedes* mosquitoes [1], dengue has established itself throughout the world’s tropical and subtropical regions in both endemic and epidemic transmission cycles, causing significant morbidity and mortality, particularly highly endemic in South-East Asia (SEA) [24–26]. However, dengue remains a seasonal disease in China, with epidemics occasionally triggered by imported dengue viruses (DENV) [27]. More than 90% of imported cases between 2005 and 2014 originated from SEA [27–31]. Following China’s economic boom in the last two decades, the number of Chinese citizens travelling abroad has increased from 5 million in 1996 to 128 million in 2015 [32]. Recent government led initiatives to further foster international trade may contribute to increased flows between SEA and China [33], which could also increase the number of importations of pathogens including DENV.

While the risk of dengue in China is apparent and growing [27], the seasonal pattern and changing risk of importation and subsequent transmission are unclear, a challenge amplified by a dearth of models for assessing seasonal risk for pathogen spread globally [18, 34]. As international travel between SEA and China by airplane is fast and common, based on the assumption that human mobility via commercial air travel is an important conduit for the spread of infectious diseases internationally, we constructed a branching process model by focusing on the seasonal and multiannual movement of DENV from the endemic countries in SEA into China via air travelers of Chinese and SEA residents between 2005 and 2015. We then retrospectively quantified and validated the seasonal risks, ranging from zero to certain (1), of DENV importation from nine SEA countries and leading to autochthonous transmission (introduced transmission) in China, identified geographic and seasonal patterns of emerging origin-destination routes, and estimated the number of imported infections in Chinese travelers and SEA residents into China. With rising concerns about global pathogen dispersal, this study provides approaches and evidence that can inform efforts to mitigate the spread of DENV and other arboviral pathogens including Zika, chikungunya, and yellow fever viruses from endemic regions.

## Methods

### Ethics statement

Ethical clearance for collecting and using secondary data in this study was granted by the institutional review board of the University of Southampton, England (No. 18152). All data were supplied and analyzed in an anonymous format, without access to personal identifying information.

### Data

#### International air travel from SEA into China

We analyzed the anonymized flight itineraries of all travelers from SEA into China between 2005 and 2015, using data obtained from the International Air Transport Association (IATA). As most travel is temporary, and local residents of SEA and Chinese travelers returning from SEA might have different risks of dengue infection and importation [23], we obtained annual statistics of the nationality of travelers entering China from the China National Tourism Administration to estimate the monthly volume of air travelers by nationality to further delineate the risk. The database is described in the Materials and Methods section in S1 Appendix.

#### Dengue incidence in SEA

The annual numbers of DENV cases were available for 17 SEA countries, but monthly data were available for nine countries (Cambodia, Laos, Malaysia, Maldives, Philippines, Singapore, Sri Lanka, Thailand, and Vietnam) to estimate the risk of dengue importation into China (Table A in S1 Appendix). To account for the substantial underreporting of dengue infections in official statistics, the monthly dengue data was further adjusted by an expansion factor (EF) and the proportion of asymptomatic infections (Table B in S1 Appendix). The EF has been commonly used to estimate the total number of dengue incidence from officially statistics [35–37]. Based on the approaches of previous studies [36, 37], the country-specific EF and its 95% uncertainty interval (UI) were estimated as the total number of dengue episodes in a specified population divided by the episodes reported, with the necessary data obtained from systematic literature review or extrapolated for countries where no empirical studies were available. We also assumed that the apparent illness represents approximately 20% (SD 10%) of all dengue infections [38, 39]. The data sources and collation are detailed in S1 Appendix.

#### Dengue incidence in China

The anonymized data of imported and autochthonous dengue cases reported in China for 2005–2015 were obtained from the China Public Health Science Data Centre (www.phsciencedata.cn). Dengue case who travelled to a dengue endemic foreign country within 15 days prior to the onset of illness was classified as imported [27]. As some cases in border regions might import to China via land travel, we excluded cases reported from cities without an airport in border areas of Yunnan, Guangxi, Tibet and Xinjiang province bordering SEA countries (Fig A in S1 Appendix).

### Analyses

#### Correlation of dengue importation and air travel

We examined the relationship between reported cases of DENV importation in China and airline travel from dengue endemic countries across SEA into China from 2005 to 2015 by using *Spearman*’s rank correlation coefficient. The wavelet analysis was conducted to characterize the periodicity of dengue transmission and the coherency of seasonal patterns between SEA and China, based on the methods described by van Panhuis et al (Materials and Methods in S1 Appendix) [40].

#### Importation and onward transmission risk

We constructed a branching process model that included both importation and onward autochthonous transmission risk estimates, with the probabilistic risk ranging from zero to one (certain). A description of the model and its structure is provided in the Materials and Methods of S1 Appendix. In brief, the probability ($p_{IMPORT}$) of at least one DENV-infected traveler importation via air travel from SEA and being infectious after arriving China was defined as a single-step *Poisson* process depending on: (1) the risk of infection in travelers during the period of stay in country with ongoing dengue virus transmission; (2) the probability of non-Chinese residents in SEA traveling into China and the probability of Chinese travelers returning to China; and (3) the duration of infection in humans as the length of the intrinsic incubation period for DENV plus the time that a person remains viremic after onset, referring to the period over which an infected person could travel and experience symptomatic disease or transmit DENV to mosquitoes [18, 34]. Furthermore, based on the *Poisson* distribution of DENV importation risk, we derived the expected monthly number of imported infections via air travel, and the *Granger* causality test [41] was used to examine the performance of estimated time series for predicting the reported time series.

The monthly probability ($p_{AUTO}$) of an introduced DENV infection from SEA leading to autochthonous transmission in China was defined as the probability in a three-step process: (1) infected airline travelers from each SEA country entering provinces or cities in China; (2) mosquitoes in China acquiring the virus from infected travelers; and (3) those infected mosquitoes infecting at least one other person in China [18, 34]. The latter two processes, human-to-mosquito and mosquito-to-human DENV transmission in China, were characterized as *Poisson* processes with means of the number of infectious mosquitoes produced per infected human and humans infected per infectious mosquito. Additionally, global maps of estimated *Aedes aegypti and Ae*. *albopictus* suitability were used to exclude areas in China unsuitable for the vector [42].

#### Parameters and model validation

For parameters describing the transmission process including the infectious period and entomological components, we used distributions informed by available data and previous analyses [18, 34]. Temperature-dependent parameters were estimated using average monthly temperature data obtained from the China Meteorological Data Service Centre. The model parameters are detailed in the S1 Appendix. The parameter distributions were incorporated into importation and introduced transmission models by sampling 10,000 sets of parameters from estimated distributions. We then computed $p_{IMPORT}$and $p_{AUTO}$with all 10,000 parameter sets and reported the median and interquartile range (IQR) to account for uncertainties. For validation, we compared the risks estimated by the models with the occurrence of imported and locally acquired DENV infections reported in the corresponding location and month in China. A receiver operating characteristic (ROC) curve and the area under the curve (AUC) were used to measure the accuracy of models.

## Results

The volume of airline travelers from 17 SEA countries into China nearly quadrupled from 3.6 million in 2005 to 13.8 million in 2015, with the most (69.3% of all 73.9 million passengers) departing from Thailand, Singapore, and Malaysia (Fig A in S1 Appendix). Nine SEA countries with available monthly dengue incidence data for risk analysis had a total of 63.4 million airline travelers (85.8% passengers from 17 SEA countries) into 165 cities in China between 2005 and 2015, including 38.7 million (61.1%) Chinese travelers and 24.7 million residents (38.9%) from nine SEA countries with Chinese increased rapidly from 1.4 million (44.8%) in 2005 to 9.5 million (79.0%) in 2015 (Figs B and C in S1 Appendix).

**Fig 1:**
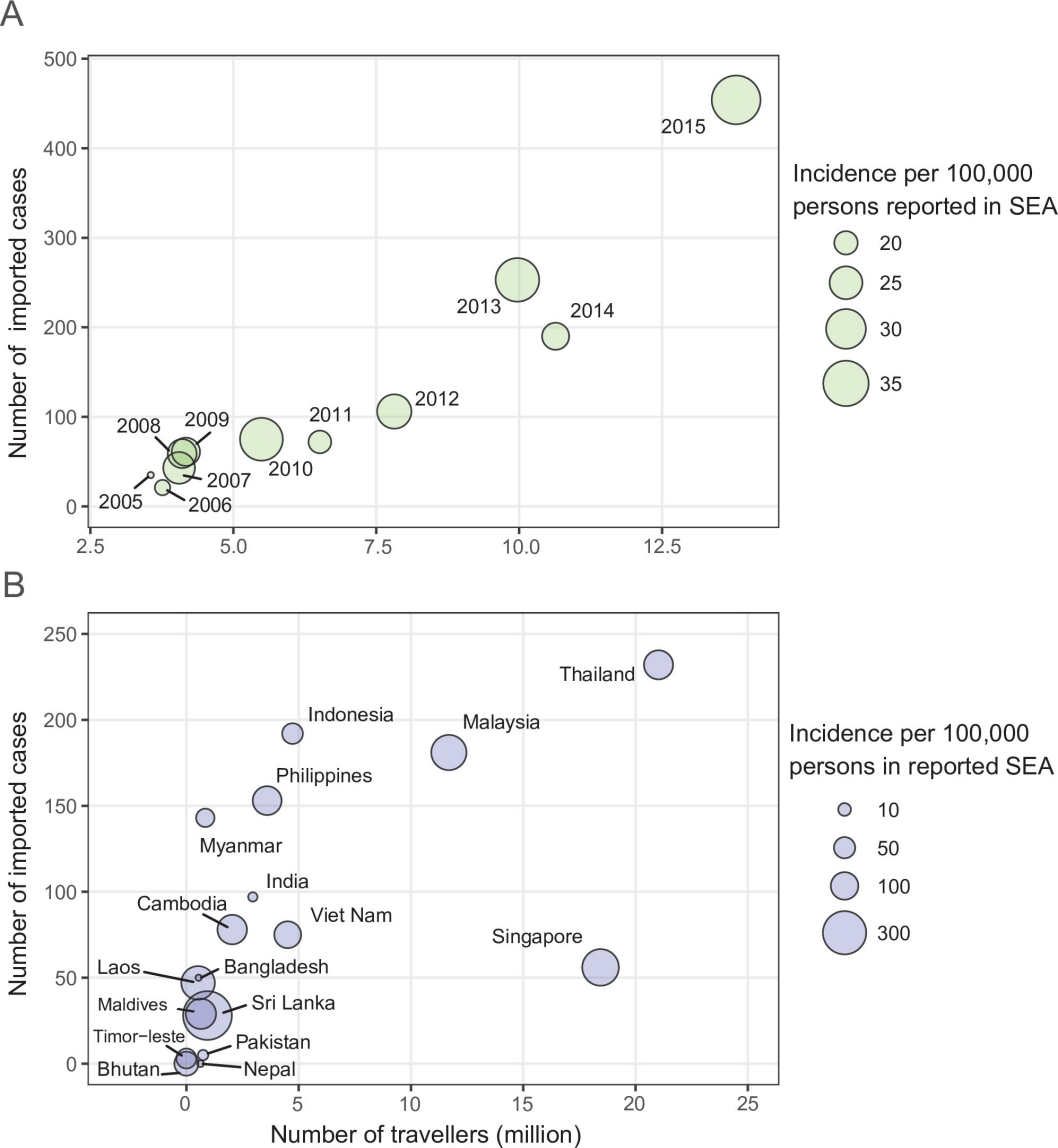
Airline travelers and dengue importation from South-East Asia into China, 2005–2015. (**A**) Yearly volume of airline travelers vs number of dengue cases imported from SEA into China. (**B**) Airline travelers vs dengue cases imported from SEA into China, aggregated by country.

**Fig 2:**
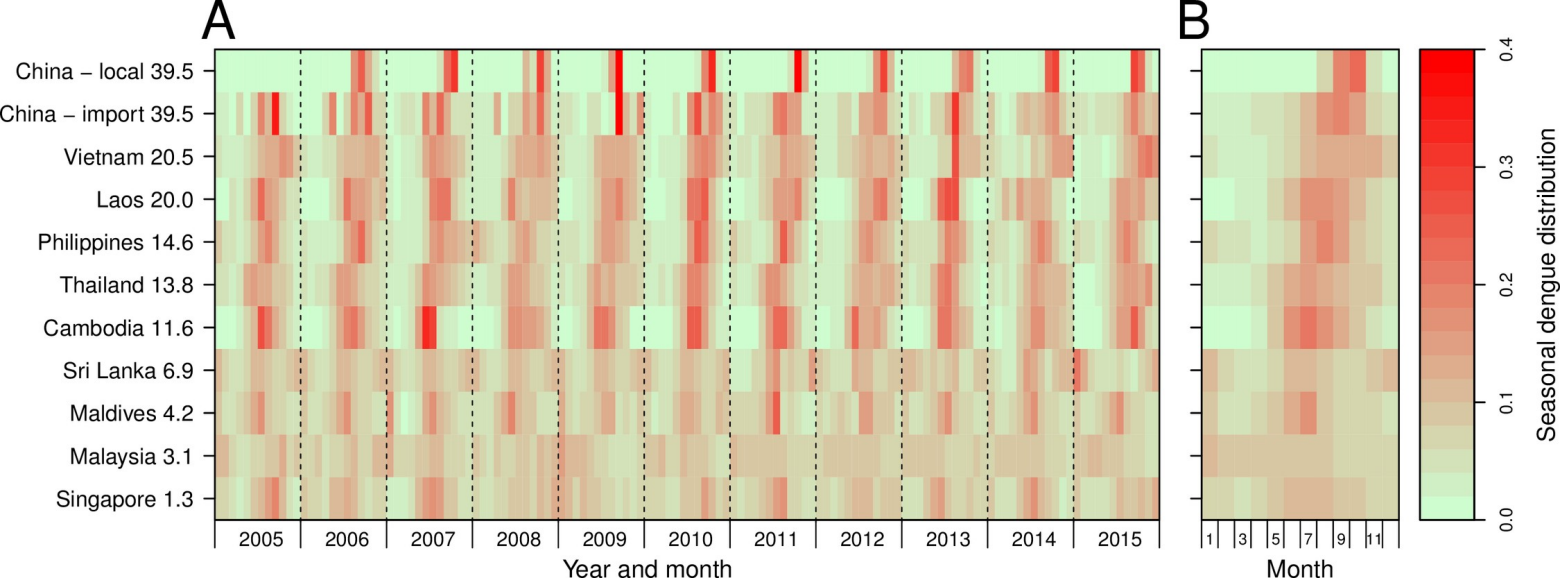
Relative seasonal variation in monthly dengue incidence for SEA countries and China, sorted by latitude, 2005–2015. **(A)** Time series of monthly dengue cases, standardized by the total number of cases reported in each year and country. **(B)** Average seasonal distribution of dengue by country, plotted as the proportion of cases reported in each week of the year from 2005 to 2015. The data of “China–import” represents the cases imported from nine SEA countries (Cambodia, Laos, Malaysia, Maldives, Philippines, Singapore, Sri Lanka, Thailand, and Vietnam) into China. The data of “China–local” represents the autochthonous cases reported in China.

Fig 1 shows the volume of travelers from SEA and the number of corresponding imported dengue cases into China have positive correlations by year and by origin (*Spearman*’s rank correlation, both p<0.001). Seasonal patterns of dengue transmission in nine countries were also seen with annual amplitude positively correlated to the latitude of each country. Furthermore, there was a significant synchrony between dengue incidence in SEA and importation to China, and the seasonal epidemics in China were also highly coherent with dengue transmission in SEA and importation into China (Fig 2 and Figs D-F in S1 Appendix).

**Fig 3:**
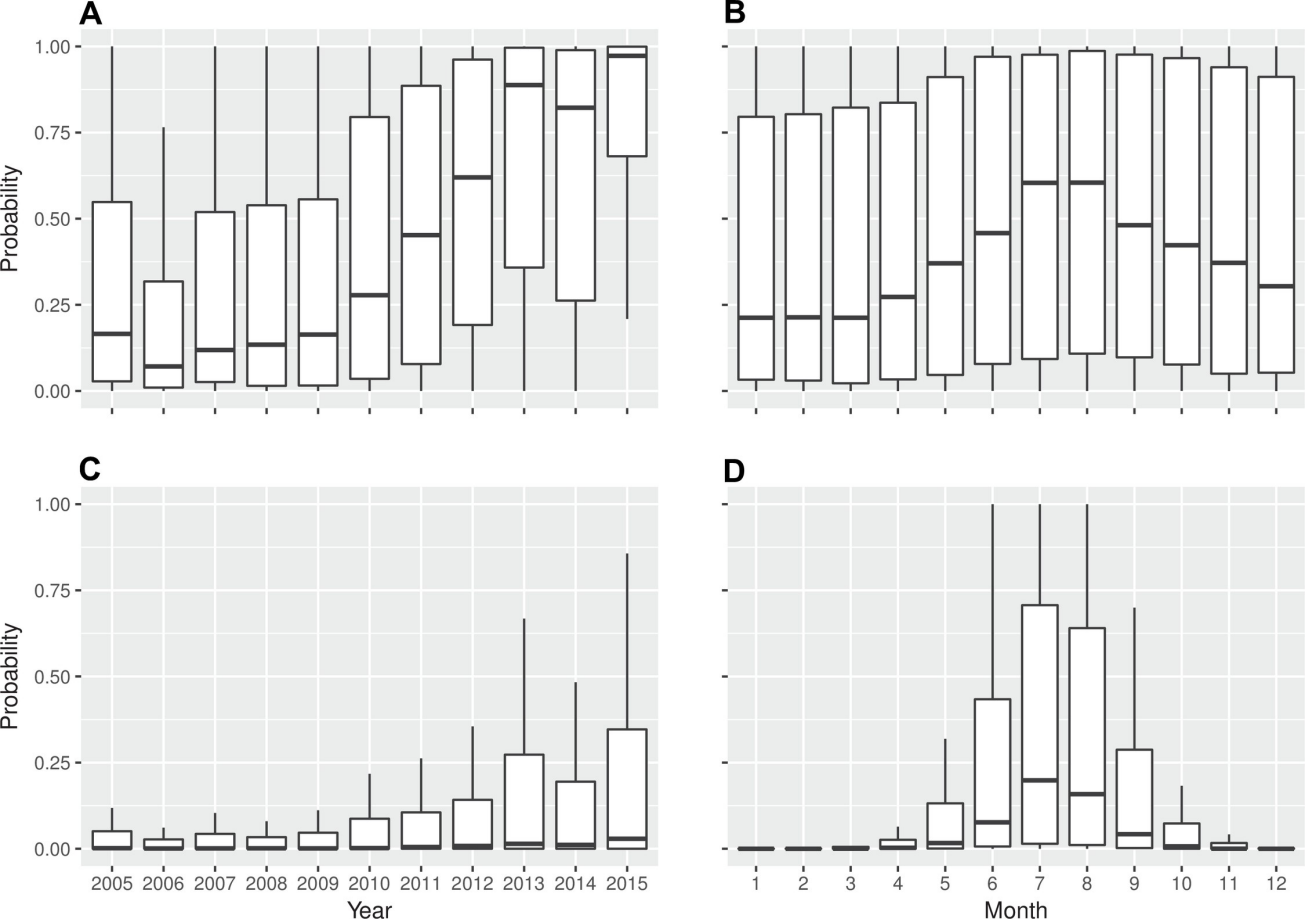
Dengue importation and introduced transmission risks from South-East Asia into provinces of mainland China, 2005–2015. **(A)** Importation risk by year. **(B)** Importation risk by month. **(C)** Introduced transmission risk by year. **(D)** Introduced transmission risk by month. The probabilistic risk presented here is the likelihood of occurrence of at least one DENV infection at provincial level. Nine countries (Cambodia, Laos, Malaysia, Maldives, Philippines, Singapore, Sri Lanka, Thailand, Vietnam) in SEA with available data of monthly DENV incidence were included here.

The monthly DENV importation risk from nine countries of SEA into a province of China have increased from a median of 0.18 (IQR 0.03–0.57) in 2005 to 0.98 (0.72–1.0) in 2015 (Fig 3, panel A). Both Chinese travelers and SEA residents contributed to increasing risk over that decade, but Chinese travelers (median 0.26, IQR 0.03–0.88) were more likely to introduce dengue into China than SEA residents (0.14, 0.02–0.56), particularly since 2011 (Figs G-I in S1 Appendix). Across all years, the lowest risk (median 0.22, IQR 0.03–0.82) was in March, and the highest (0.65, 0.12–1.0) was in August when 23 cities (13.9% of 165 cities) had an average risk greater than 0.5 between 2005 and 2015 (Fig 3, panel B and Figs J and K in S1 Appendix).

**Fig 4:**
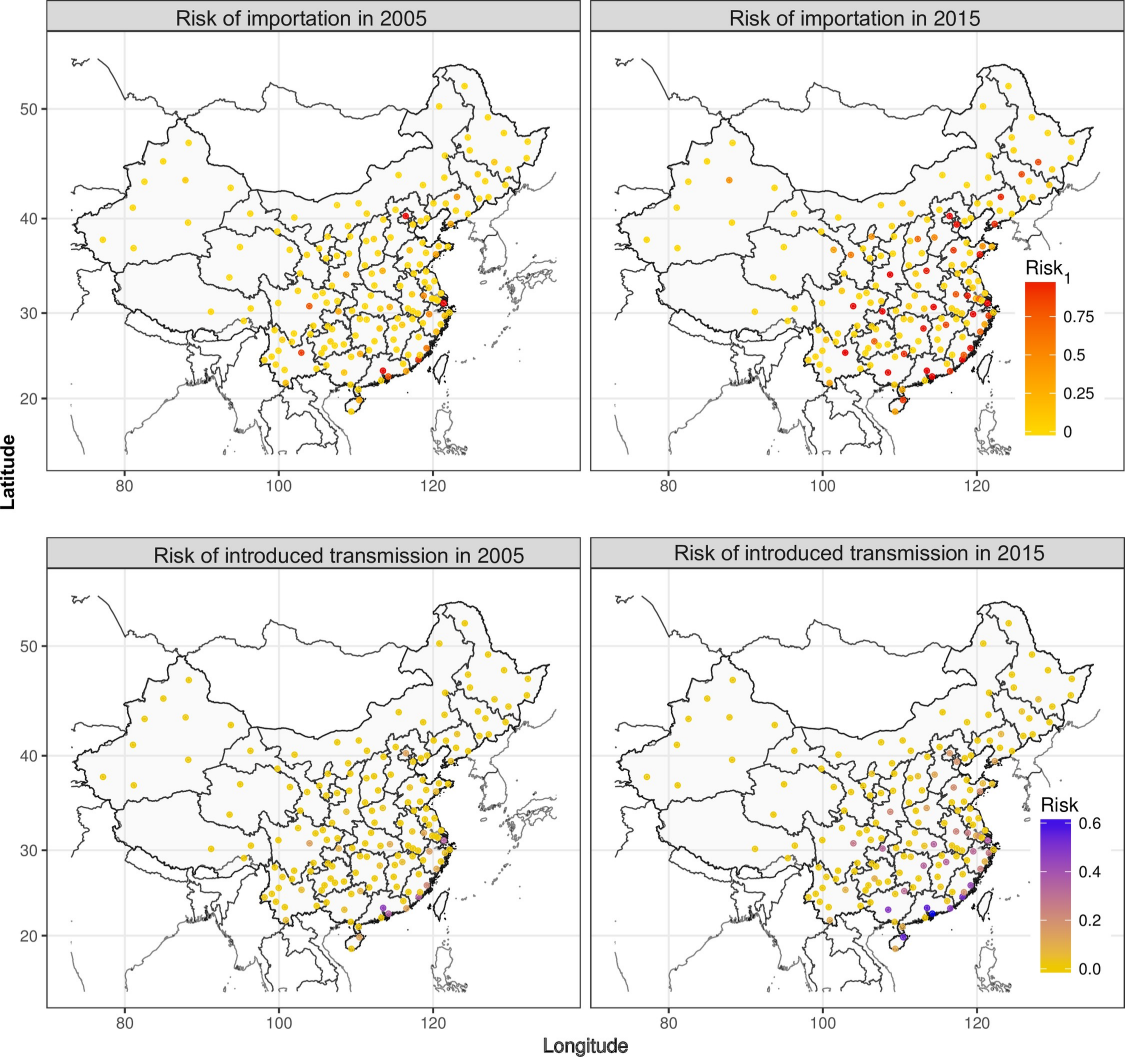
Geographic risks of dengue importation and introduced transmission from South-East Asia into cities of mainland China in 2005 and 2015. The probabilistic risks (0–1) were estimates for travelers from nine SEA countries (Cambodia, Laos, Malaysia, Maldives, Philippines, Singapore, Sri Lanka, Thailand, and Vietnam) into 165 cities in China.

**Fig 5:**
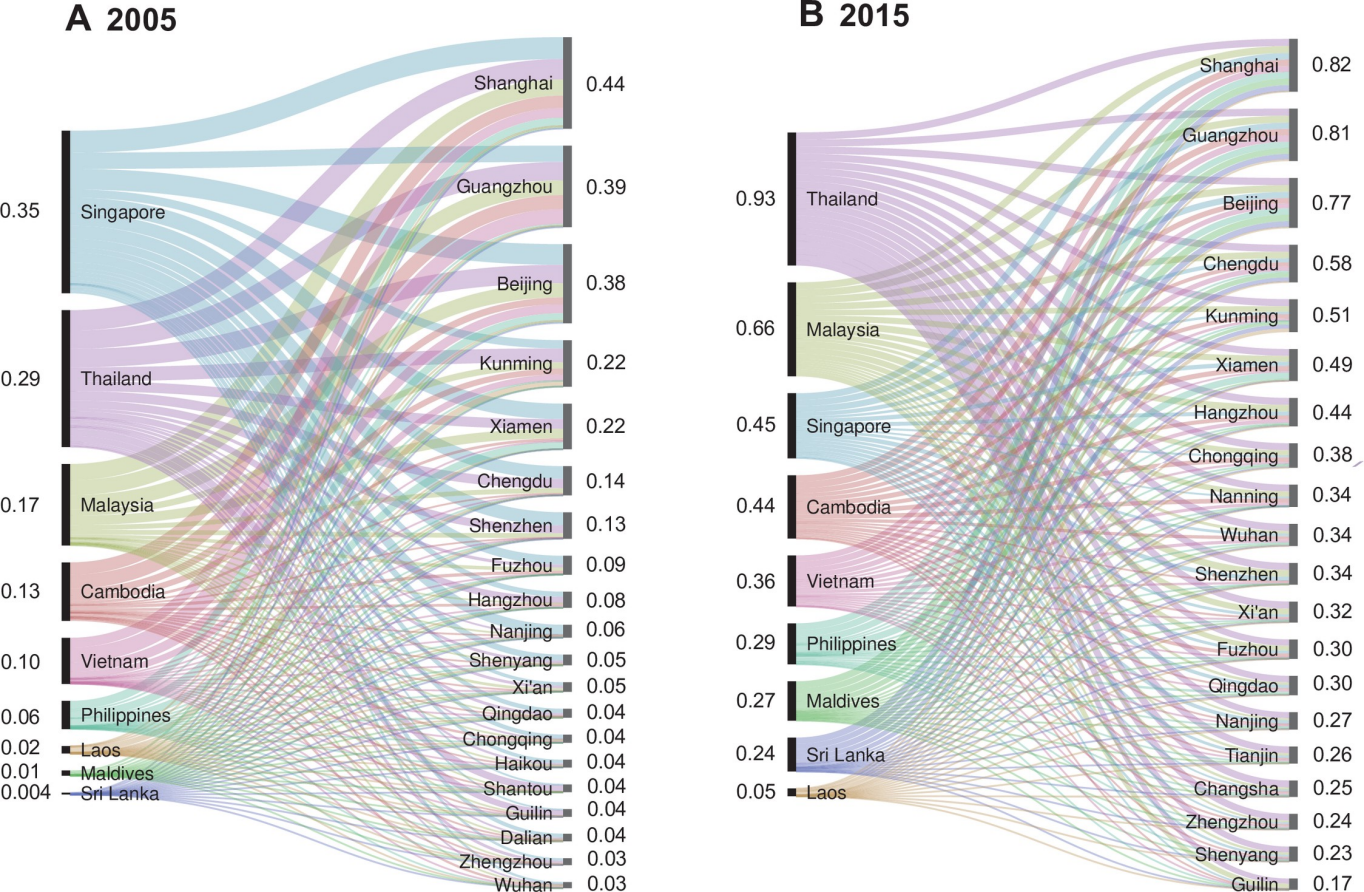
Origin-destination routes of dengue importation from SEA into top 20 high-risk cities of China in 2005 and 2015. For 2005 **(A)** and 2015 **(B)**, the lines indicate the most likely routes of DENV importation with thickness scaled to the estimate importation risk from the lowest (thinnest) to highest (thickest) for each panel. The numbers on the left indicate the average monthly probability of exporting at least one infected case from each origin to any of the cities of China and the numbers on the right, the average monthly probability of importation to each destination.

The percentage of cities with a median importation risk higher than 0.5 increased from 4.8% (8/165) in 2005 to 21.8% (36/165) in 2015 with most emerging destinations in central and western China (Fig 4), and cities with a median probability of risk greater than 0.5 due to Chinese travelers increased from 7 to 35, versus 5 to 18 for SEA residents (Fig L in S1 Appendix). Thailand, Malaysia and Singapore were consistently amongst the locations with the highest risk for DENV importation into China; while Sri Lanka and Maldives were emerging as important origins due to the increasing travel, particularly in Chinese (Fig 5 and Figs M and N in S1 Appendix). Meanwhile, among the 1485 routes from nine SEA countries to 165 cities of China, those with a median risk higher than 0.5 rose from 15 (1.0%) in 2005 to 84 (5.7%) in 2015.

**Fig 6:**
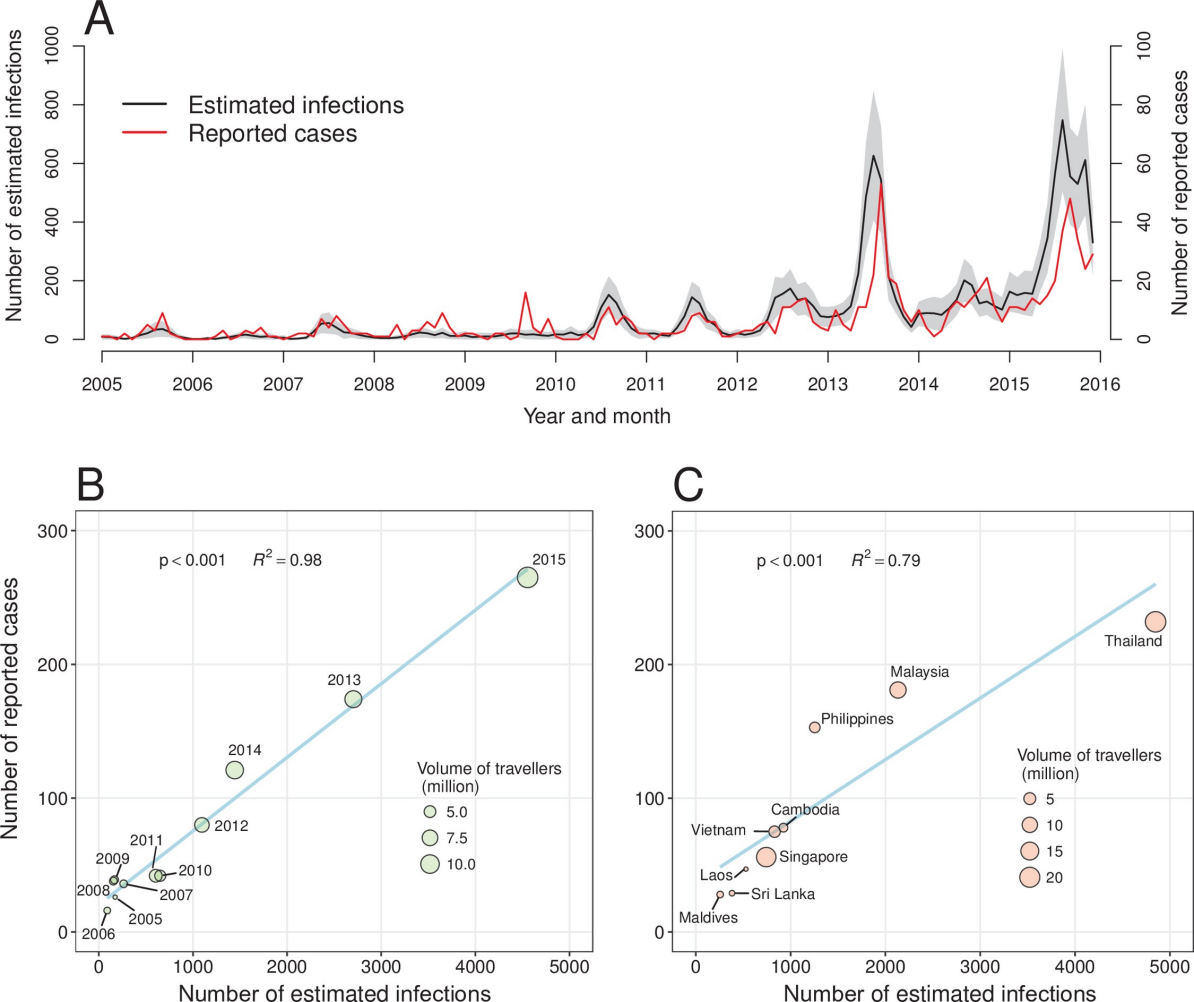
Estimates of imported DENV infections from nine SEA countries into China, 2005–2015. (**A**) Time series of estimated infections in airline travelers, with 95% UI of estimates, and the reported cases imported from SEA. (**B**) Estimated infections vs reported cases by year. (**C**) Estimated infections vs reported cases by country. We estimated all symptomatic and asymptomatic infections that can possibly introduce transmission. In (**B**) and (**C**), symbol size is proportional to the volume of travelers from SEA into China, and blue solid lines represent linear regression fit, with p values and adjusted R-square values on the graphs.

A total of 11,901 infections (95% UI 6923–16,917) via air travel was estimated to import from nine SEA countries into China between 2005 and 2015, which was 13.5 times (7.8–19.2) of the 879 imported cases reported in dengue surveillance system of China. The estimates had positive correlations with the reported numbers by month and country, and by nationality (Fig 6 and Figs O and P in S1 Appendix). Furthermore, the estimated time series with a one-month lag could significantly predict the numbers of cases reported in surveillance (F = 203.7, p<0.001) (Fig Q in S1 Appendix).

The probability of DENV introduced local transmission from nine SEA countries into China also rose, with a median risk increasing from 0.10 (IQR 0.01–0.30) in August 2005 to 0.56 (IQR 0.21–0.91) in August 2015 at provincial level (Fig 3, panel C). Significant seasonal variation was evident, with high risk during the warm months between May-October, but very low risk in other months (Fig 3, panel D). Compared to cities with intensive importation in cold regions of northern China, e.g. Beijing and Shenyang, the introduced risks in the lower latitude cities, e.g. Guangzhou, Shenzhen and Haikou, were much higher and extended over longer time periods (Fig 4 and Fig K in S1 Appendix). The countrywide change in the probability of introduced transmission between 2005 and 2015 led to a much larger population being at risk: Guangzhou, Shanghai and Xiamen with 32 million people were the only three cities with a risk greater than 0.5 in August 2005, while there are 102 million people in 10 cities (Guangzhou, Shanghai, Fuzhou, Xiamen, Shenzhen, Hangzhou, Haikou, Nanning, Wuhan, Changsha and Chongqing) with the same risk in August 2015 (Fig R in S1 Appendix).

The dynamics of dengue in SEA, the volume, demography and immunity level of airline travelers, and the environmental suitability of DENV local transmission in China have been changing the high-risk routes for importation and introduced transmission (Figs S and T in S1 Appendix). For instance, the Maldives-Guangzhou, Philippines-Fuzhou and Malaysia-Hangzhou routes have had increasing risks since 2005, and cities in central China and middle coastal regions, e.g. Hangzhou, Chengdu and Wuhan, are emerging as destinations with an increasing risk of introduced transmission. Additionally, compared to the monthly occurrence of cases reported at the provincial level in China, the ROC curves showed our model performed robustly with an AUC of 0.86 for importation risk estimates and 0.92 for introduced transmission estimates (Fig U in S1 Appendix). Moreover, the importation risk estimates for SEA residents had a slight better performance than for Chinese travelers (AUC 0.91 vs 0.86).

## Discussion

Being able to identify areas at risk for introduction and spread of pathogens in a timely manner is critical for situational awareness and for tailoring strategies for preparedness and response, e.g. allocation of finite health and human resources [27]. In this study, we constructed a branching process modelling framework to elucidate seasonal probability of international spread of mosquito-borne viral disease from endemic countries in SEA via air travel. We have identified the emerging origins in SEA and locations in China that are most susceptible to dengue importation and onward transmission, and we also revealed the seasonal patterns and increasing risks in routes of DENV spread by air travel over a decade. The spatiotemporal heterogeneities of DENV importation risk have also been seen in the travelers of Chinese and SEA residents. The risk of introduced transmission from particular routes identified can be used to inform efforts to prevent and control the spread of DENV, and are particularly important for currently neglected, high-risk locations, i.e. Chengdu, Wuhan and Hangzhou. Moreover, with the increasing risk of dengue importation and transmission from SEA, China can also be the source of exportation, and this was amply shown in the introduction of dengue from Guangzhou into Japan resulting in a small outbreak in Tokyo [43].

The geographic, historical, and cultural ties between SEA countries and China, as well as increasing economic and tourism links, has contributed to the growing travel volume. We demonstrate here the epidemiological significance of this travel in the context of DENV importation from these countries into China over a decade. Compared to SEA residents travelling into China, the accelerated growth in the volume of international Chinese travelers over time has also facilitated increased DENV importation from SEA. For instance, the growth in risk of dengue importation from Sri Lanka since 2010 can likely be attributed to the increasing investment and workers from China [44], while the rising risk from the Maldives is probably related to increasing numbers of Chinese tourists [32].

The megacities in China, e.g. Beijing, Shanghai and Guangzhou, each regional aviation hubs, have consistently received large volumes of international air passengers, leading to high risks of dengue importation from SEA. However, the rapid growth of travel abroad for tourism, business and migrant workers from cities in central and southwest China is also sufficient to cause substantial risk of importation. A similar pattern has been described for malaria importation from Africa and SEA into these areas [45]. The increase in imported DENV from SEA has also increased subsequent transmission risk in China, with Guangdong, Yunnan and Fujian provinces frequently reporting outbreaks following dengue importation throughout the last decade. Meanwhile, other provinces (e.g. Henan, Shandong and Shanghai) have reported autochthonous cases of dengue for the first time [27, 46]. The increasing importation risk, together with increasing temperatures and the spatial spread of *Ae*. *aegypti* [47], are all contribute to increased risk of introduced transmission and the potential for year-round autochthonous transmission of DENV and other flaviviruses in several tropical and subtropical regions of China (e.g. Hainan, Guangdong and Yunnan). The variation in DENV serotypes introduced from different origins over time is especially relevant considering the potential for adverse effects from dengue haemorrhagic fever after infection with a different serotype of DENV [27].

The number of imported cases reported in surveillance systems could be predicted by the estimated time series with one-month lag, which might be due to the longer period of travel, and the delay identification and reporting of imported infections by the routine surveillance. The gaps between the estimates and reported numbers found in this study also highlight the needs to improve the capacity of surveillance systems and formulate strategies to mitigate these imported contagions, and public health authorities and partners in areas with huge volume of imported infections and high risk of autochthonous transmission should consider implementing appropriate actions at an early stage of potential seasonal transmission. These could include health education in Chinese travelers and early identifying the infections in entry points, and improve the capacity of surveillance, vector control, laboratory diagnosis, and clinical management.

However, the risk of introduction is a more complex function that reflects more than travel volumes [48, 49], e.g. the incidence of the disease in the country of disembarkation, the probability of being infected/viremic at the time of travel and arrival in the destination country, the duration of viremia, the presence of favorable conditions (vectors and seasonality) in the destination. Therefore, our findings must be considered in the context of several assumptions and data limitations. First, the quality of incidence data on dengue incidence in SEA and China likely varies due to differences in surveillance systems including case definitions, reporting methods, availability of healthcare and laboratory diagnosis, under reporting, and the completeness and accuracy of data reported. Second, the risk of dengue infection in SEA was assumed to be identical across each country, without considering the immunity of different serotypes in Chinese travelers and SEA residents. Third, we only estimated the risk of the Chinese and SEA residents, but the actual nationalities in travelers might be much complex, not only the Chinese and SEA residents, but also the residents from other countries passing through SEA on their way to China. Fourth, we regarded *Ae*. *albopictus* as an equally competent vector to *Ae*. *aegypti* for DENV, with similar temperature dependency and extrinsic incubation period. Fifth, our estimates did not address variability in the public health and health-care capacity and resources for different years and locations in China and SEA countries in response to dengue. Furthermore, due to the availability of monthly disease incidence in SEA countries and the absence of monthly travel data by land and water from SEA into China and within China, we only estimate the seasonal risk of introduction through air travel for nine SEA countries. Therefore, the total risk of dengue introductions from all SEA countries into China must be underestimated in this study. To solve these problems in future studies, the monthly dengue incidence for all countries could be estimated by mathematical models based on epidemiological and entomological parameters and climate data, and the seasonal and multiannual cross-border population movements could be further estimated by gravity-type spatial interaction models or using novel sources of data, e.g. mobile phone data or social media data [50–52].

Nonetheless, the models and findings presented here leverage previous work suggesting that a probabilistic model of pathogen spread over a heterogeneous network by multiple populations could capture most of the information in much complex stochastic simulation models [18, 34]. Moreover, our retrospective validation showed that the predicted seasonal risk of DENV into China coincided with a surge in the number of imported cases and volume of airline travelers arriving into China from SEA countries with reported dengue virus activity. Our model framework is sufficiently flexible to incorporate new forms of data and adapt to different vector-borne diseases. Moreover, it may be used to project into the future given different scenarios and to quantify the effects of different control methods. It also highlights the need for high-quality, accessible travel and surveillance data at national, regional, and global levels. As shown here, travel dynamics have a direct and drastic impact on regional and global infectious disease dynamics and having accessible data to assess those risks in real time can support appropriate risk assessment and prevention, and control activities.

## Supporting information

S1 Appendix The appendix includes the materials and methods of data collation and analysis, tables A and B, and figs A–U. (PDF)
