# Supplementary material for: Seasonal and interannual risks of dengue introduction from South-East Asia into China, 2005-2015
Source: PLoS Negl Trop Dis. 2018 Nov 9;12(11):e0006743. doi: 10.1371/journal.pntd.0006743 (PMC6248995; doi:10.1371/journal.pntd.0006743)
Supplement: S1 Appendix — (PDF) [file pntd.0006743.s001.pdf]

Supporting Information for

Seasonal and interannual risks of dengue introduction from South-East Asia into China, 2005-2015

Shengjie Lai, Michael A Johansson, Wenwu Yin, Nicola A Wardrop, Willem G van Panhuis, Amy Wesolowski, Moritz U G Kraemer, Isaac I Bogoch, Dylain Kain, Aidan Findlater, Marc Choisy, Zhuojie Huang, Di Mu, Yu Li, Yangni He, Qiulan Chen, Juan Yang, Kamran Khan, Andrew J Tatem, Hongjie Yu

This file includes:

Materials and Methods

Tables A and B

Figs. A to U

References

Materials and Methods

Air travel from South-East Asia into China

Airline ticket sales and flight itinerary data were obtained from the International Air Transport Association (IATA) to analyze the travel pattern of all passengers initiating trips from any airport in (South-East Asia) SEA with a destination in China between January 2005 and December 2015. These itineraries included data on the initial airports of embarkation in SEA and all direct and non-direct flight connections up to the travelers' final airport destination in China. Previous studies suggested that residents in endemic countries and travelers from non-endemic countries into endemic areas might have various risks of mosquito-borne infections, due to the duration of stay, immunity, and locations, etc. (*1*). To delineate the potential different risks of dengue infection in SEA and importation from SEA into China by local residents of SEA and Chinese travelers returning from SEA between 2005 and 2015, we estimated the monthly volume of Chinese travelers ($T^{c}_{i,s,m}$) and SEA residents ($T^{f}_{i,s,m}$) as

$$T^{f}_{i,s,m} = \sigma T_{i,s,m}$$

$$T^{c}_{i,s,m} = (1-\sigma)T_{i,s,m}$$

where σ is the proportion of SEA residents in all travelers from each SEA country into China. To estimate monthly σ, we first calculated country-specific annual σ using annual statistics of the numbers of SEA residents traveling into China and Chinese travelers returning into China by SEA country, obtained from the China National Tourism Administration (www.cnta.gov.cn/zwgk/lysj/). To account for the variations between monthly and annual proportions, and between routes, we then assumed that monthly σ had an SD of 10% annual proportion and sampled 10,000 sets for dengue importation risk estimation.

Dengue incidence in SEA

The official statistics of dengue cases reported by 17 SEA countries (Cambodia, Bangladesh, Bhutan, India, Indonesia, Laos, Malaysia, Maldives, Myanmar, Nepal, Pakistan, Philippines, Singapore, Sri Lanka, Thailand, Timor-Leste, and Vietnam) in 2005-2015 were collated from Project Tycho (www.tycho.pitt.edu) and other publicly

available sources, including the World Health Organization and regional offices and the ministries of health in individual countries. We also searched PubMed for relevant articles in English published from January 1, 2005 to December 31, 2016 with the query "((Dengue[Title]) AND "country name"[Title]) AND ("2005/01/01"[Date - Publication]: "2016/12/31"[Date - Publication])", and the search engine (www.google.com) was queried with keywords, such as "Dengue", year and name of country in English, to identify the aggregated data of dengue epidemics from English language news releases. The data sources and collation were detailed in Table A.

As dengue infection by any dengue virus can produce a wide spectrum of illness, with most infections are asymptomatic or subclinical, cases reported in passive surveillance systems may be under-recognized. To account for the substantial underreporting of dengue incidence in official statistics, the monthly dengue data reported by nine countries was further adjusted as

$$I_{s,m} = \frac{I_{s,m}^{R} F_s}{\mu}$$

where $I_{s,m}^{R}$ is the monthly number of cases reported by country *s* in month *m*, F_s is an expansion factor (EF) of each country, and μ is the proportion of symptomatic cases in dengue infections. The EF has been commonly used to estimate the total number of dengue incidence from officially statistics (*2-4*). We estimated the country-specific EF and its 95% UI (Table B) based on Undurraga et al (*4*), who derived EFs through a systematic analysis of published studies that reported original, empirically derived EFs or the necessary data to obtain them (*3*). For countries where no empirical studies were available, data were extrapolated based on the statistically significant inverse relationship between an index of a country's health system quality and its observed reporting rate. Based on previous evidence, we assumed that the apparent illness (μ) represents approximately 20% (SD 10%) of all dengue infections (*5, 6*). Additionally, to compute the incidence rate for each country, the country- and year-specific population data of SEA were obtained from the World Bank (data.worldbank.org/), and the population data in China were obtained from the National Bureau of Statistics of China.

Seasonality and synchrony of dengue transmission between SEA and China

We used wavelet analysis to characterize the periodicity of dengue transmission and the coherency of seasonal patterns between SEA and China, based on the methods described by van Panhuis et al (*7*). First, the monthly incidence rates for countries in SEA and China were de-trended by subtracting fitted values from the observed values by a linear model, and centred (discounted the mean) and reduced (divided by the SD) to *z* scores to increase cross-country comparability. Then, the continuous wavelet transforms with the complex Morlet wave using a nondimensional frequency $\omega_0 = 6$ and a periodicity step size δ_j of 0.25 on a linear scale were conducted for each country to enable the extraction of a high resolution of the periodicity scale (1-mo intervals) and obtain phase angles to represent epidemic timing (*7-9*). Finally, we measured wavelet coherency between dengue transmission time series in SEA and dengue importation time series from SEA into China. The wavelet coherency uses wave transforms of two time-series to indicate their localized phase relationship in a time–frequency spectrum. Statistical significance of wavelet coherency was tested using Monte Carlo methods (n = 600) (*7, 10*).

Importation and onward autochthonous transmission risk estimates

To quantify the seasonality and the risk of DENV importation and spread within China, we constructed a branching process model (Figure G) that included both importation and onward autochthonous transmission risk estimates, with the probabilistic risk ranging from zero to one.

Quantifying importation risk

The probability (p_{IMPORT}) of at least one DENV-infected traveler importation via air travel from SEA and being infectious after arriving China was defined as a single-step *Poisson* process depending on: (1) the risk of infection in country with ongoing dengue virus transmission, characterized as the number of dengue infections among local SEA residents ($I_{s,m}^{f}$) and among Chinese travelers ($I_{s,m}^{c}$) during the period of stay (*C*) in SEA; (2) the probability of non-Chinese residents in SEA traveling into China ($p_{i,s,m}^{f}$) and the probability of Chinese travelers returning to China ($p_{i,s,m}^{c}$); and (3) the duration of infection in humans (*D*) as the length of the intrinsic incubation period for DENV plus

the time that a person remains viremic after onset, referring to the period over which an infected person could travel and experience symptomatic disease or transmit DENV to mosquitoes (*11, 12*). For all Chinese travelers and SEA residents traveling into China, the risks of DENV importation from an endemic country *s* into a location *i* of China in month *m* were estimated as

$$p_{IMPORT}(i, \mathrm{s}, m) = 1 - e^{-\lambda_{i,s,m}}$$

$$p_{IMPORT\_c}(i, \mathrm{s}, m) = 1 - e^{-\lambda^{c}_{i,s,m}}$$

$$p_{IMPORT\_f}(i, \mathrm{s}, m) = 1 - e^{-\lambda^{f}_{i,s,m}}$$

where

$$\lambda_{i,s,m} = \lambda^{c}_{i,s,m} + \lambda^{f}_{i,s,m}$$

$$\lambda^{c}_{i,s,m} = I^{c}_{s,m} D p^{c}_{i,s,m}$$

$$\lambda^{f}_{i,s,m} = I^{f}_{s,m} D p^{f}_{i,s,m}$$

As the mean intrinsic incubation period for DENV is approximately 6 days and the infectious period post-onset is 3–5 days (*13*), we assumed *D* is 10 days (SD 1 day) in this model. For the Chinese population, $p^{c}_{i,s,m}$ is ~1 because the average duration of stay for Chinese residents in other countries is approximately 7 days, so virtually all Chinese travelers are expected to return to China within one month. For the local population in SEA, $p^{f}_{i,s,m}$is the number of local SEA residents travelling to China divided by the total population of the respective country.

To estimate the $I^{c}_{s,m}$ and $I^{f}_{s,m}$, we first estimated the total number of person days for Chinese travelers ($A_{s,m}$) and locals ($B_{s,m}$) in SEA as

$$A_{s,m} = C \sum_{i} T^{c}_{i,s,m}$$

$$B_{s,m} = F_m G_{s,m} - C \sum_{i} T^{f}_{i,s,m}$$

where $T^{c}_{i,s,m}$ and $T^{f}_{i,s,m}$ are the numbers of Chinese travelers and local travelers respectively, F_m is the number of days in each month, $G_{s,m}$ is the total yearly population of each country. *C* is the duration of stay in SEA for Chinese or in China for residents from SEA, parameterized as 7 days (SD 1 day) based on the statistics from the China

National Tourism Administration.

We then estimated daily prevalence of DENV infection in this combined population as

$$E_{s,m} = \frac{I_{s,m}D}{A_{s,m} + B_{s,m}}$$

where $I_{s,m}$ is the number of dengue cases adjusted by underreporting in country *s* and month *m*. Given the high dengue burden and ecological suitability of vectors in SEA (*14, 15*), we assumed that travelers and locals had equal exposure risk in SEA and that travelers from/to other locations (not between *s* and *i*) in a country of SEA just replaced each other.

Based on the daily risk of being infected estimated above, and the total person days of Chinese travelers and residents in SEA, the probability of travel, and the immunity of travelers, $\lambda_{i,s,m}^{c}$ and $\lambda_{i,s,m}^{f}$ can be further derived as

$$\lambda_{i,s,m}^{c} = E_{s,m}T_{i,s,m}^{c}R_{i}^{c}C$$

$$\lambda_{i,s,m}^{f} = E_{s,m}B_{s,m}R_{s}^{f}\frac{T_{i,s,m}^{f}}{G_{s,m}}$$

where R_{i}^{c} and R_{s}^{f} are the proportions of susceptible population in Chinese and SEA travelers, respectively, to adjust for population immunity. As the average yearly incidence rate of dengue reported in China between 1990 and 2014 was only 2.2 cases per one million residents (*16*), we assumed all Chinese travelers were susceptive to dengue viruses from 2005 to 2015 (R_{i}^{c}~1). Given the high force of infection in the nine study countries, new infections are concentrated among children (who presumably travel less) and most adults are immune for at least one serotype of DENV (*6*), we then assumed that the susceptible proportion, R_{s}^{f}, for SEA travelers was 20% (SD 10%).

The monthly probabilities of dengue importation by origin and destination were estimated as follows:

$$p_{IMPORT}(s,m) = 1 - \prod_{i \in I} e^{-\lambda_{i,s,m}}$$

$$p_{IMPORT}(i,m) = 1 - \prod_{s \in S} e^{-\lambda_{i,s,m}}$$

Estimating imported infections

Based on the *Poisson* distribution of DENV importation risk and $\lambda_{i,s,m}$ defined above, we further derived the expected number of infections imported by Chinese and SEA residents from country *s* into a location *i* of China in month *m*. The expected value and variance of a *Poisson*-distributed random variable are both equal to λ, and the estimates included all symptomatic and asymptomatic infections that can possibly introduce transmission. The mean and 95% UI of the estimates of infected travelers, by Chinese and SEA residents, were computed using the distributions of importation risk from all 10,000 parameter sets, and the estimates were compared with the number of imported cases reported in China.

Quantifying Introduced transmission risk

The monthly probability (p_{AUTO}) of an introduced DENV infection from SEA leading to autochthonous transmission in China was defined as the probability in a three-step process: (1) infected airline travelers from each SEA country entering provinces or cities in China; (2) mosquitoes in China acquiring the virus from infected travelers; and (3) those infected mosquitoes infecting at least one other person in China (*11, 12*). The introduced transmission risk in a location (*i*) of China and month *m* was estimated as

$$p_{AUTO}(i,m) = 1 - \prod_{s \in S} e^{\lambda_{i,s,m} e^{R_{0i,m}^{HM}\left(e^{-R_{0i,m}^{MH}}-1\right)}-1}$$

where $\lambda_{i,s,m}$ used the estimates for p_{IMPORT}. The human-to-mosquito and mosquito-to-human DENV transmission processes in China were characterized as *Poisson* processes with means of the number of infectious mosquitoes produced per infected human ($R_{0i,m}^{HM}$) and humans infected per infectious mosquito ($R_{0i,m}^{MH}$), defined as

$$R_{0i,m}^{HM} = \varphi_{i,m}\alpha\beta_{HM}V\gamma_{i,m}$$

$$R_{0i,m}^{MH} = \alpha\beta_{MH}L_{i,m}$$

where $\varphi_{i,m}$ refers to the number of mosquitoes per person in a location *i* and month

m, *α* is the daily biting rate, β_{HM} refers to the probability of transmission given an infectious blood meal, and β_{MH} is the probability of transmission given an infectious bite, V refers to the number of days a human is infectious, $\gamma_{i,m}$ is the proportion of mosquitoes surviving the extrinsic incubation period, and $L_{i,m}$ is the number of days an infectious mosquito survives. Given the airport mainly serviced for the local regions, we assumed that the travelers with DENV infections arrived at the cities or provinces of destinations without further cross-city or province movement during infectious period.

Introduced risk model parameterization

To estimate the number of infectious mosquitoes produced per infected human ($R_{0i,m}^{HM}$) and the number of humans infected per infectious mosquito ($R_{0i,m}^{MH}$) in introduced transmission model, the parameters were defined as below:

Human infectious period (*V*): As patients are typically viremic for 3-5 days, and mosquitoes can become infected by dengue virus (DENV) when biting viremic hosts, we assumed *V* was 4 days (SD 1 day).(*13*)

Mosquito biting rate (*α*): A detailed study of blood meals suggests that *Ae. aegypti* feed 0.63–0.76 times per day.(*17*) We assumed that *Ae. albopictus* behaved similarly and *α* used a mean of 0.7 blood meals per day (SD 0.05) as previous study.(*12*)

Human-to-mosquito transmissibility (β_{HM}): β_{HM} is the probability of a mosquito acquiring DENV while feeding on a viremic human. As we estimated the human infectious period based on the 50% infectious dose, we assumed that β_{HM} was 0.5 (SD 0.1).

Mosquito-to-human transmissibility (β_{MH}): Transmissibility of DENV from infected mosquitoes to humans is unknown, yet it is likely less than 100%. We assumed that the probability was 0.5 (SD 0.1).

Extrinsic incubation period (*EIP*): *EIP* is the period in the mosquito after acquiring the virus and prior to being able to transmit the virus. Temperature-specific data for DENV are limited to the range of 26–30°C.(*18*) We assumed that the average *EIP* at 28°C (EIP_{28}) was 6 days (SD 2 days) and the relationship with temperature (*T*) was $\beta_T = -0.08$ (SD 0.02).(*18*) We sampled from both distributions to estimate the mean

EIP for each location as:

$$EIP = e^{(\log EIP_{28})e^{\beta_T (T-28)}}$$

Mosquito survival (*γ* and *L*): The mortality of *Aedes* mosquitoes in the field depends on many factors including weather and species.(*19*) The mean mortality for *Ae. albopictus* and *Ae. aegypti* across temperature was estimated by fitting a polynomial curve, $\mu(T)$, to the relationship between temperature and average daily mortality.(*12, 19*)

$$\mu(T) = 0.3967 - 0.03912T + (2.442 \times 10^{-3})T^2 - (7.479 \times 10^{-5})T^3 + (9.298 \times 10^{-7})T^4$$

We then assumed that the month- and location-specific average mosquito lifespan (*L*) was $1/\mu(T)$ days with a SD of 2 days. To incorporate the uncertainty associated with both mosquito mortality and the *EIP*, the proportion of mosquitoes surviving in the *EIP* was then calculated as:

$$\gamma = e^{-EIP/L}$$

Mosquito density (*φ*): We assumed that there were 1–3 mosquitoes per person, an average of 2 (SD 1) under ideal weather conditions. To account for the population-wide effects of increased mortality at temperature extremes, we estimated the density was proportional to the minimum mortality:

$$\varphi_{i,m} = \varphi\left(\frac{L_{i,m}}{\max L}\right)$$

where *φ* is the density under ideal weather conditions, $L_{i,m}$ is a location- and month-specific, temperature-dependent lifespan, and max *L* is the maximum mean lifespan of vectors.

Tables

Table A. Data source and collation of annual and monthly dengue incidence data in South-East Asian countries, 2005-2015.

Country	Source of annual incidence	Source of monthly incidence
**Cambodia**	1. The annual numbers of dengue cases in 2005-2010 were obtained and aggregated from the Project Tycho (www.tycho.pitt.edu).<br><br>2. The data in 2011-2015 were obtained from Cambodia Early Warning System in the Communicable Disease Control Department of the Ministry of Health (www.cdcmoh.gov.kh/surveillance/camewarn).	1. The monthly numbers of dengue cases in 2005-2010 were obtained from the Project Tycho (www.tycho.pitt.edu).<br><br>2. The weekly data in 2011-2012 and 2014-2015 were obtained from the reports of the Communicable Disease Control Department of the Ministry of Health (www.cdcmoh.gov.kh/surveillance/camewarn) then aggregated to monthly incidence.<br><br>3. The monthly incidence of dengue in 2013 was estimated by: the total annual number of cases in 2013 × the proportion of cases in the same month of other years between 2005 and 2015.
**Bangladesh**	1. The annual data in 2005-2012 were collated from the WHO Regional Office for South-East Asia (www.searo.who.int/entity/vector_borne_tropical_diseases/data/graphs.pdf?ua=1).<br><br>2. The data in 2013-2015 were obtained from the Annual Health Bulletin published by the Ministry of Health and Family Welfare (www.dghs.gov.bd/index.php/en/data); and<br><br>3. Sharmin S, Viennet E, Glass K, Harley D. The emergence of dengue in Bangladesh: epidemiology, challenges and future disease risk. Transactions of the Royal Society of Tropical Medicine and Hygiene 2015; **109**(10): 619-27.	Unavailable
**Bhutan**	1. The annual data in 2005-2012 were collated from the WHO Regional Office for South-East Asia (www.searo.who.int/entity/vector_borne_tropical_diseases/data/graphs.pdf?ua=1).	Unavailable

Country	Source of annual incidence	Source of monthly incidence
	2. The yearly data in 2013-2015 were obtained from the online Annual Health Bulletins published by the Ministry of Health (www.health.gov.bt/publications/annual-health-bulletins).	
**India**	1. The annual data in 2005-2012 were collated from the WHO Regional Office for South-East Asia (www.searo.who.int/entity/vector_borne_tropical_diseases/data/graphs.pdf?ua=1).<br><br>2. The data in 2013-2015 were obtained from the national Vector Borne Disease Control Programme of the Ministry of Health and Family Welfare (nvbdcp.gov.in/DENGU1.html).	Unavailable
**Indonesia**	1. The annual data in 2005-2012 were collated from the WHO Regional Office for South-East Asia (www.searo.who.int/entity/vector_borne_tropical_diseases/data/graphs.pdf?ua=1).<br><br>2. The data in 2013-2015 were obtained from the Annual Indonesia Health Profile (www.depkes.go.id/folder/view/01/structure-publikasi-pusdatin-profil-kesehatan.html), and reports published by the Ministry of Health (www.depkes.go.id/article/view/15011700003/demam-berdarah-biasanya-mulai-meningkat-di-januari.html).	Unavailable
**Laos**	1. The annual numbers of dengue cases in 2005-2010 were obtained and aggregated from the Project Tycho (www.tycho.pitt.edu).<br><br>2. The data in 2011-2015 were obtained from the National Surveillance Weekly Report published by the National Center for Laboratory and Epidemiology of the Ministry of Health (www.ncle.gov.la/epidemiology/national-surveillance-weekly-report/).	1. Monthly numbers of dengue cases in 2005-2010 were obtained from the Project Tycho (www.tycho.pitt.edu).<br><br>2. Annual data in 2011-2015 were obtained and aggregated from the National Surveillance Weekly Report published by the National Center for Laboratory and Epidemiology of the Ministry of Health (www.ncle.gov.la/epidemiology/national-surveillance-weekly-report/).
**Malaysia**	1. The annual numbers of dengue cases in 2005-2010 were obtained and aggregated from the Project Tycho (www.tycho.pitt.edu).	1. The monthly numbers of dengue cases in 2005-2010 were obtained and aggregated from the Project Tycho (www.tycho.pitt.edu).

Country	Source of annual incidence	Source of monthly incidence
	2. The data in 2011-2015 were obtained from the weekly Press Release of Dengue Fever Situation published by the Ministry of Health Malaysia (www.moh.gov.my/english.php/database_stores/store_view/17).	2. The monthly data in 2011-2015 were obtained from the weekly Press Release of Dengue Fever Situation published by the Ministry of Health Malaysia (www.moh.gov.my/english.php/database_stores/store_view/17).
**Maldives**	1. The annual data in 2005-2012 were collated from the WHO Regional Office for South-East Asia (www.searo.who.int/entity/vector_borne_tropical_diseases/data/graphs.pdf?ua=1).<br>2. The annual numbers of cases in 2013-2015 were obtained from the annual Maldives Health Statistics or Profile published on the official website of the Ministry of Health (www.health.gov.mv/Downloads).	1. The monthly numbers of cases in 2005-2014 were obtained from the annual Maldives Health Statistics published on the website of the Ministry of Health (www.health.gov.mv/Downloads).<br>2. The monthly incidence of dengue in 2015 was estimated by: the total annual number of cases in 2015 × the proportion of cases in the same month in previous years of 2005-2014.
**Myanmar**	1. The annual data in 2005-2012 were collated from the WHO Regional Office for South-East Asia (www.searo.who.int/entity/vector_borne_tropical_diseases/data/graphs.pdf?ua=1).<br>2. The data in 2013 were obtained from: Ngwe Tun MM, Kyaw AK, Makki N, et al. Characterization of the 2013 dengue epidemic in Myanmar with dengue virus 1 as the dominant serotype. Infection, genetics and evolution: journal of molecular epidemiology and evolutionary genetics in infectious diseases 2016; **43**: 31-7.<br>3. The incidence data in 2014 were obtained from the report of the Ministry of information of Myanmar (www.moi.gov.mm/moi:eng/?q=news/29/06/2015/id-4205)<br>4. The data in 2015 were collated from the press release (outbreaknewstoday.com/myanmar-health-minister-dengue-cases-increasing-since-april-more-than-7000-reported-27057/ and<br>frontiermyanmar.net/en/the-dreaded-dengue-on-the-rise).	Unavailable

Country	Source of annual incidence	Source of monthly incidence
**Nepal**	1. The annual data in 2005-2012 were collated from the WHO Regional Office for South-East Asia (www.searo.who.int/entity/vector_borne_tropical_diseases/data/graphs.pdf?ua=1) and checked with the data in the publication:<br><br>Subedi D, Taylor-Robinson AW. Epidemiology of dengue in Nepal: History of incidence, current prevalence and strategies for future control. Journal of vector borne diseases 2016; **53**(1): 1-7.<br><br>2. The annual numbers of dengue cases in 2013-2015 were obtained from the Annual Report 2015/2016 published by the Department of Health Services, Ministry of Health (dohs.gov.np/wp-content/uploads/2017/06/DoHS_Annual_Report_2072_73.pdf).	Unavailable
**Pakistan**	1. Annual numbers of dengue cases in 2006-2010 were obtained from WHO Regional Office for the Eastern Mediterranean (www.emro.who.int/surveillance-forecasting-response/outbreaks/dengue-fever-in-pakistan.html).<br><br>2. Data in 2005 and 2011 were extracted from a paper: Rasheed SB, Butlin RK, Boots M. A review of dengue as an emerging disease in Pakistan. Public health 2013; **127**(1): 11-7.<br><br>3. The data in 2012 were extracted from a study of Principal Component Analysis to Explore Climatic Variability and Dengue Outbreak in Lahore (pjsor.com/index.php/pjsor/article/viewFile/686/362).<br><br>4. Data in 2013 were collated from the Weekly Epidemiological Bulletin of the WHO country office for Pakistan bulletin (www.emro.who.int/images/stories/pakistan/documents/pak_documents/DEWS/Weekly-Epidemiological-Bulletin-52-01012014.pdf?ua=1) and the publication: Wesolowski A, Qureshi T, Boni MF, et al. Impact of human mobility on the emergence of dengue epidemics in	Unavailable

Country	Source of annual incidence	Source of monthly incidence
	Pakistan. Proceedings of the National Academy of Sciences of the United States of America 2015; **112**(38): 11887-92.<br><br>5. Annual data in 2014-2015 were extracted from press release (aaj.tv/2016/01/32600-dengue-cases-registered-129-people-died-in-three-years/).	
**Philippines**	1. The annual numbers of dengue cases in 2005-2010 were obtained and aggregated from the Project Tycho (www.tycho.pitt.edu).<br><br>2. The annual data in 2011-2015 were obtained from the statistics of disease surveillance (www.doh.gov.ph/statistics) and weekly Dengue Morbidity Reports (www.doh.gov.ph/notifiable_diseases) published by the Department of Health.	1. Monthly numbers of dengue cases in 2005-2010 were obtained and aggregated from the Project Tycho (www.tycho.pitt.edu).<br><br>2. The data in 2011-2015 were obtained from the statistics of disease surveillance (www.doh.gov.ph/statistics) and weekly Dengue Morbidity Reports (www.doh.gov.ph/notifiable_diseases) published by the Department of Health, then aggregated into monthly data.
**Singapore**	1. The annual numbers of dengue cases in 2005-2010 were obtained and aggregated from the Project Tycho (www.tycho.pitt.edu).<br><br>2. The annual data in 2011-2015 were obtained from the Weekly Infectious Diseases Bulletin published by the Ministry of Health (www.moh.gov.sg/content/moh_web/home/statistics/infectiousDiseasesStatistics/weekly_infectiousdiseasesbulletin.html)	1. The monthly data in 2005-2010 were obtained and aggregated from the Project Tycho (www.tycho.pitt.edu).<br><br>2. The data in 2011-2015 were obtained from the Weekly Infectious Diseases Bulletin published by the Ministry of Health (www.moh.gov.sg/content/moh_web/home/statistics/infectiousDiseasesStatistics/weekly_infectiousdiseasesbulletin.html), then aggregated into monthly data.
**Sri Lanka**	1. The annual data in 2005-2012 were collated from the WHO Regional Office for South-East Asia (www.searo.who.int/entity/vector_borne_tropical_diseases/data/graphs.pdf?ua=1).<br><br>2. The annual numbers of dengue cases in 2013-2015 were obtained and aggregated from the monthly data released by the Epidemiology Unit of the Ministry of Health (www.epid.gov.lk/web/index.php?option=c	1. The monthly numbers of dengue cases in 2010-2015 were obtained from the monthly data released by the Epidemiology Unit of the Ministry of Health (www.epid.gov.lk/web/index.php?option=com_casesanddeaths&Itemid=448&lang=en#).<br><br>2. The weekly numbers of dengue cases in 2006-2009 were obtained from the Weekly Epidemiological Report released by the Epidemiology

Country	Source of annual incidence	Source of monthly incidence
	om_casesanddeaths&Itemid=448&lang=en#).	Unit of the Ministry of Health (www.epid.gov.lk/web/index.php?option=com_content&view=article&id=163&Itemid=450&lang=en), then were aggregated into monthly data.<br>3. The monthly incidence of dengue in 2005 was estimated by: the total annual number of cases in 2005 × the proportion of cases in the same month in other years of 2006-2015.
**Thailand**	1. The annual data in 2005-2012 were collated from the WHO Regional Office for South-East Asia (www.searo.who.int/entity/vector_borne_tropical_diseases/data/graphs.pdf?ua=1).<br>2. The data in 2013-2015 were obtained from<br>the online Summary of Dengue Annual Situation released by the Bureau of Epidemiology, Department of Disease Control, Ministry of Public Health (www.boe.moph.go.th/boedb/surdata/disease.php?dcontent=old&ds=66).	1. The monthly numbers of dengue cases in 2005-2010 were obtained and aggregated from the Project Tycho (www.tycho.pitt.edu).<br>2. The monthly data in 2011-2015 were obtained and aggregated from the online Summary of Dengue Annual Situation released by the Bureau of Epidemiology, Department of Disease Control, Ministry of Public Health (www.boe.moph.go.th/boedb/surdata/disease.php?dcontent=old&ds=66).
**Timor-Leste**	1. The annual data in 2005-2012 were collated from the WHO Regional Office for South-East Asia (www.searo.who.int/entity/vector_borne_tropical_diseases/data/graphs.pdf?ua=1).<br>2. The data in 2013 were extracted from the website of the Ministry of Health (www.moh.gov.bn/SitePages/Information%20on%20Dengue.aspx).<br>3. The data in 2014 were extracted from the press release (modasys.net/3g/index.php/news-events/around-brunei/local-news/26251-significant-drop-in-dengue-cases-since-2014.html).<br>4. The annual data in 2015 was the average number of cases in 2012-2014.	Unavailable
**Vietnam**	1. The annual numbers of dengue cases in 2005-2010 were obtained and aggregated from the Project Tycho (www.tycho.pitt.edu).	1. The monthly numbers of dengue cases in 2005-2010 were obtained and aggregated from the Project Tycho (www.tycho.pitt.edu).

Country	Source of annual incidence	Source of monthly incidence
	2. The annual data in 2011-2015 were extracted from the Dengue Situation Updates published by the WHO Regional Office for Western Pacific Region (www.wpro.who.int/emerging_diseases/documents/Dengue_Archives/en/).	2. The monthly data in 2011-2015 were extracted from the Dengue Situation Updates published by the WHO Regional Office for Western Pacific Region (www.wpro.who.int/emerging_diseases/documents/Dengue_Archives/en/).

Table B. The expansion factors by country.

Country	Expansion factor (EF)	95% uncertainty interval (UI)
Cambodia	12.9	3.9 – 29.3
Laos	11.3	8.8 – 15.9
Malaysia	3.8	2.5 – 6.2
Maldives	7.7	4.2 – 11.2
Philippines	7.0	6.2 – 7.9
Singapore	4.1	1.0 – 4.9
Sri Lanka	21.5	8.3 – 45.6
Thailand	8.5	8.0 – 12.5
Vietnam	5.8	5.4 – 6.7

Note: Estimates for expansion factors based on the estimates and approaches from Undurraga et al (*4*).

Figures

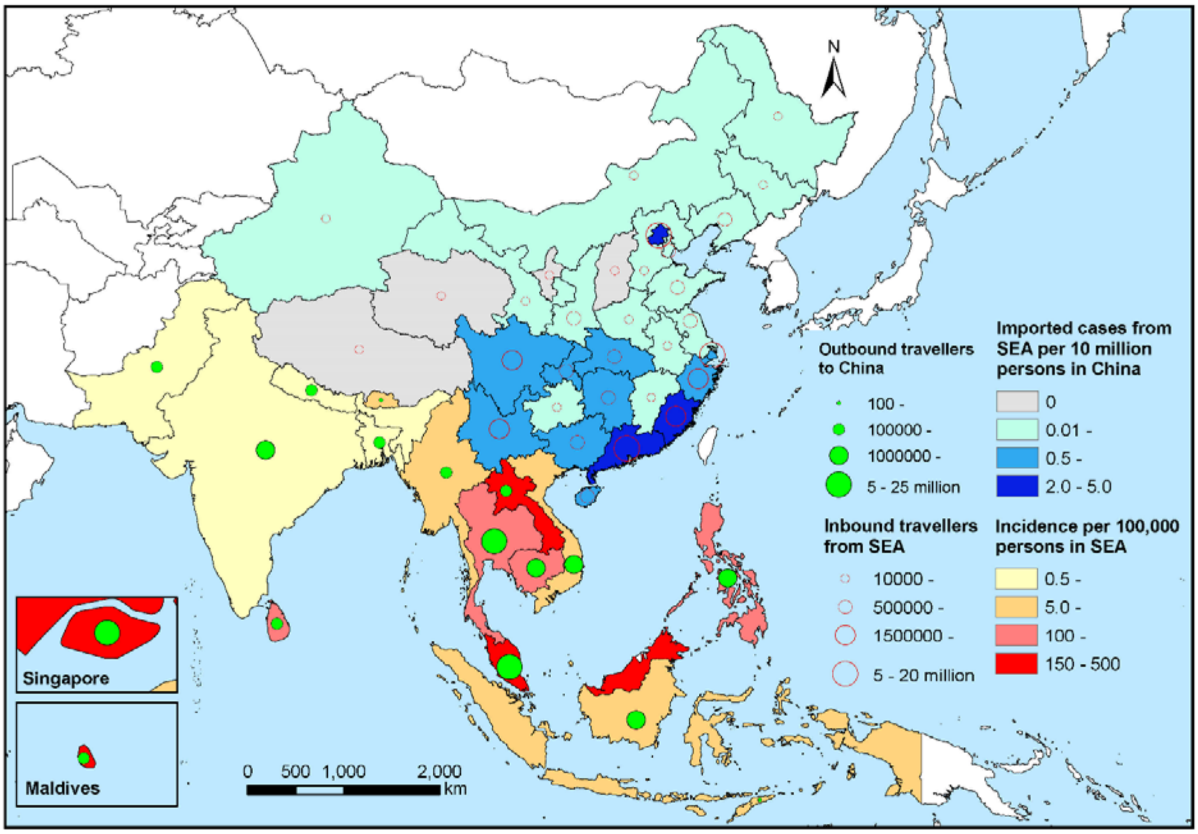

Figure A. Geographic range of dengue incidence reported in South-East Asia, airline travellers and imported dengue cases from South-East Asia into 31 provinces of mainland China.

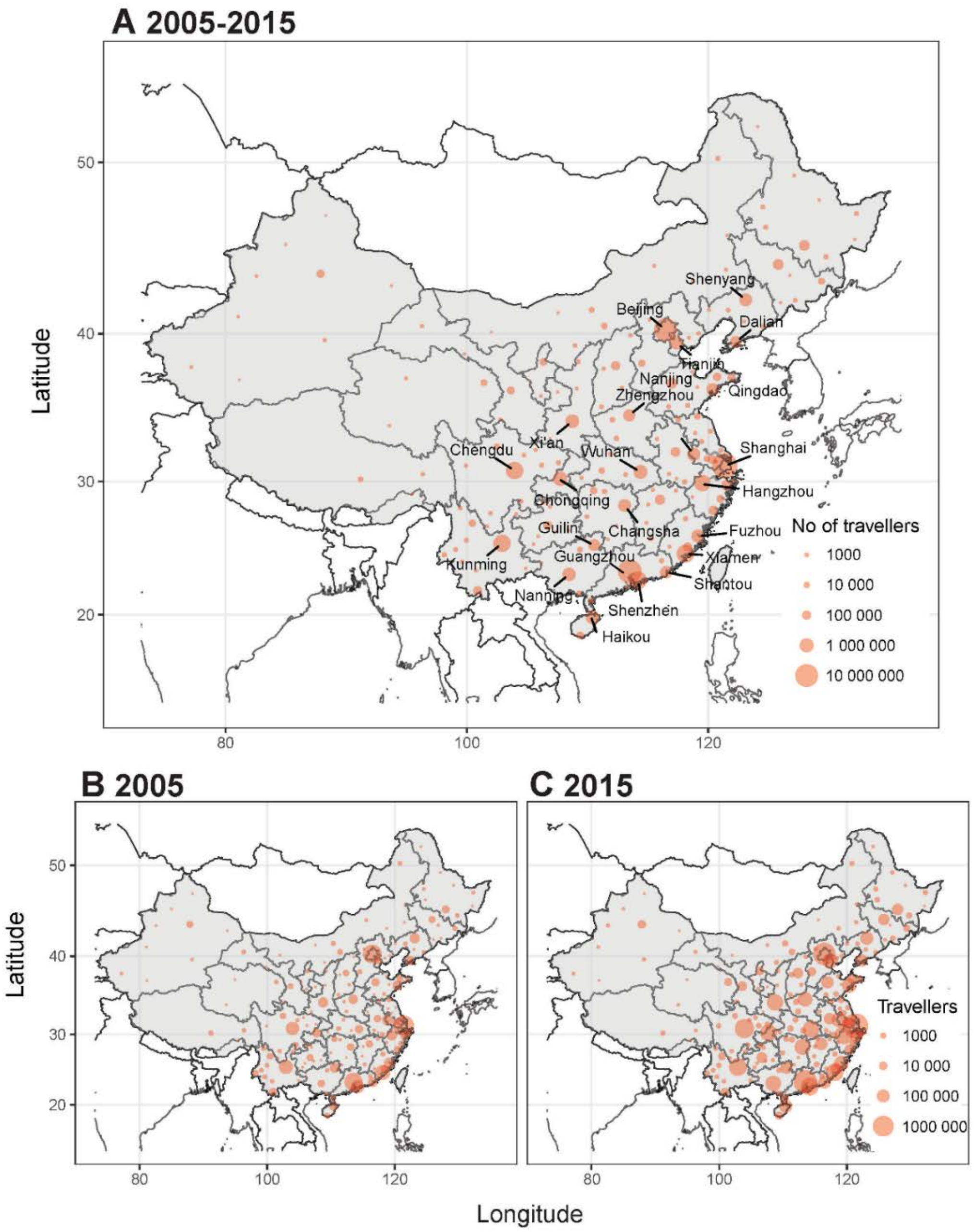

Figure B. Volume of airline travellers by destination from nine countries of South-East Asia into cities of mainland China, 2005-2015. (A) Total volume of travellers from 2005 to 2015. (B) Volume of travellers in 2005. (C) Volume of travellers in 2015. Nine countries (Cambodia, Laos, Malaysia, Maldives, Philippines, Singapore, Sri Lanka, Thailand, and Vietnam) with available data of monthly DENV incidence were included as origins.

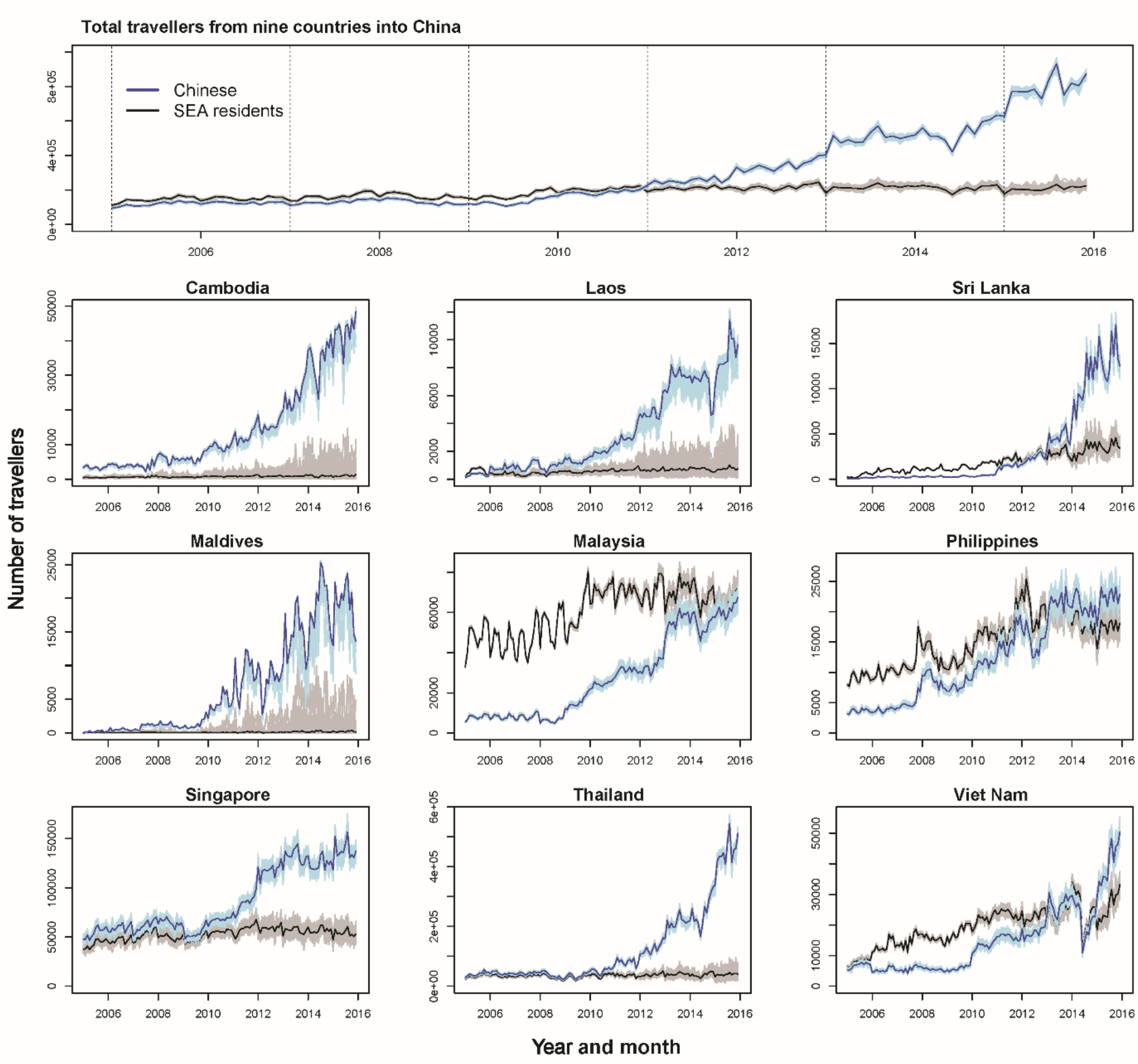

Figure C. The volume of airline travelers from nine countries of South-East Asia into China, 2005-2015. The mean and 95% UI of monthly number of travelers by nationality are presented. Nine countries with available monthly dengue incidence data for importation risk analysis are included here.

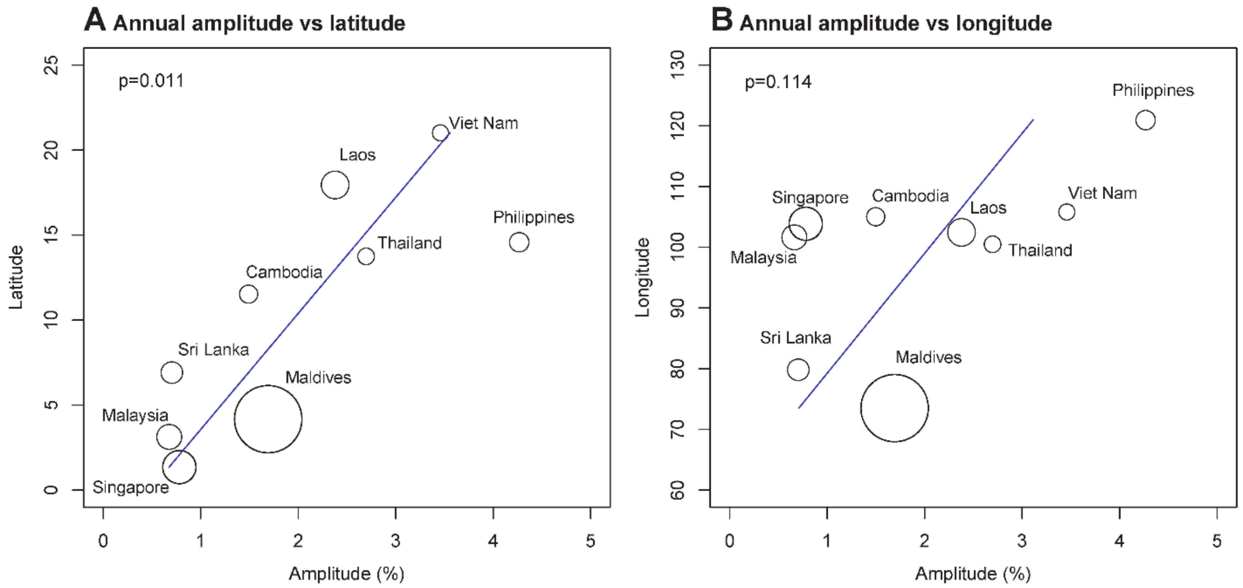

Figure D. Latitudinal and longitudinal gradients in periodicity of dengue in nine SEA countries, 2005-2015. (A) Amplitude of the annual periodicity vs the latitude of capital city of each country. (B) Amplitude of the annual periodicity vs longitude. Symbol size is proportional to the dengue incidence rate in each country. Blue solid lines represent linear regression fit. p values are given on the graphs.

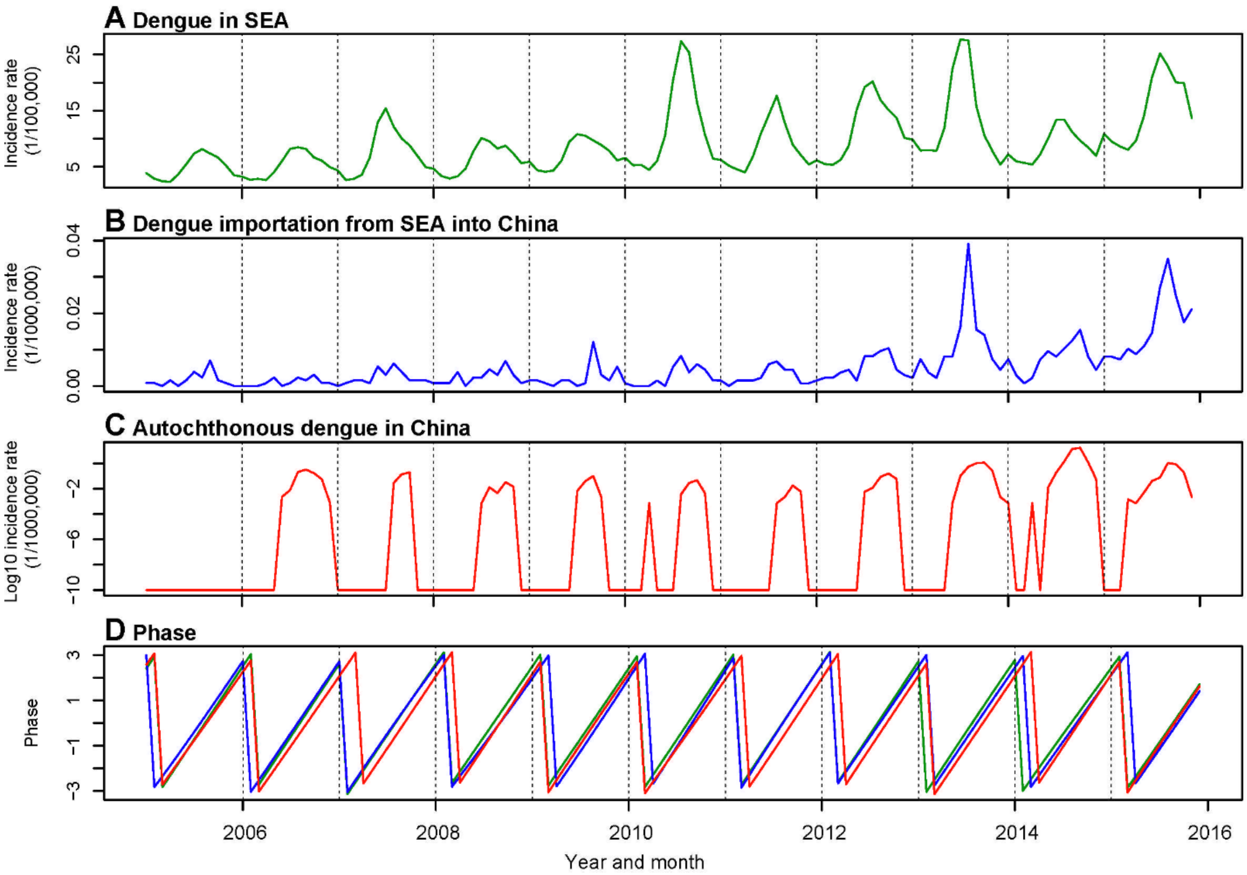

Figure E. Time series and phase of monthly dengue incidence in SEA and China. (A) Incidence rates (per 100,000 people) of dengue reported in nine SEA countries (Cambodia, Laos, Malaysia, Maldives, Philippines, Singapore, Sri Lanka, Thailand, and Vietnam). (B) Incidence rates (per 1,000,000 people) of dengue importation reported in China from nine SEA countries. (C) The log-transformed incidence rates (per 1,000,000 people) of autochthonous dengue reported in China. The number of 10^{-10} was added into each number of the time series to replace zero before log-transforms. (D) Phase angles of three time series above by wavelet transforms and reconstruction with annual dengue cycles. Green line represents dengue incidence rate in SEA; Blue represents dengue importation from SEA into China; Red line represents autochthonous dengue incidence rate (log10-transformed) in China.

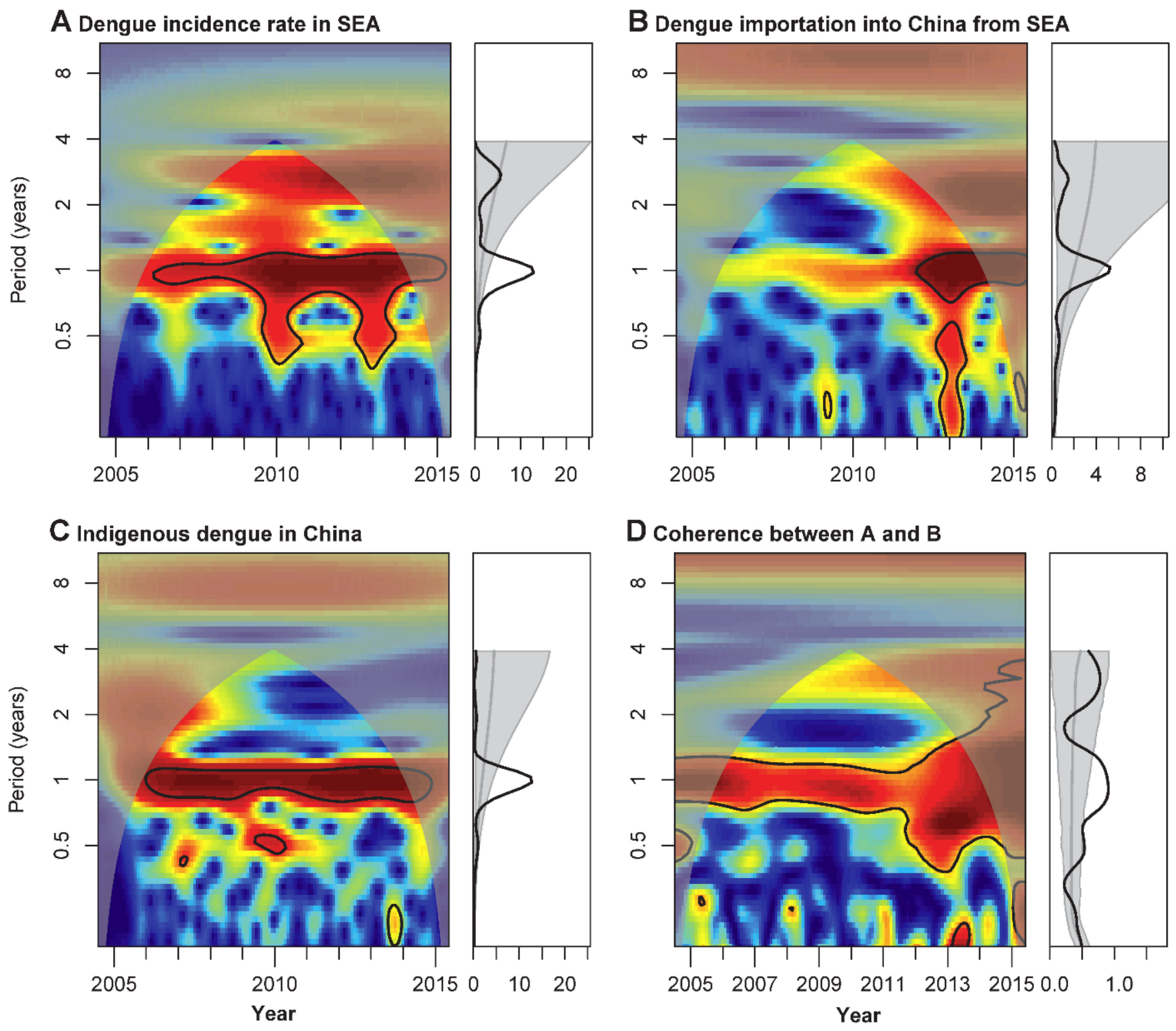

Figure F. Spectrums and coherency of dengue incidence in nine SEA country and China by wavelet transforms. (A) Spectrum of incidence rates (per 100,000 people) of dengue reported in nine SEA countries. (B) Spectrum of incidence rates (per 1,000,000 people) of dengue importation reported in China from nine SEA countries. (C) Spectrum of the log-transformed incidence rates (per 1,000,000 people) of indigenous dengue reported in China. The number of 10^{-10} was added into each number of the time series to replace zero figures before log-transforms and wavelet transforms. (D) Synchrony between the spectrums of A and B.

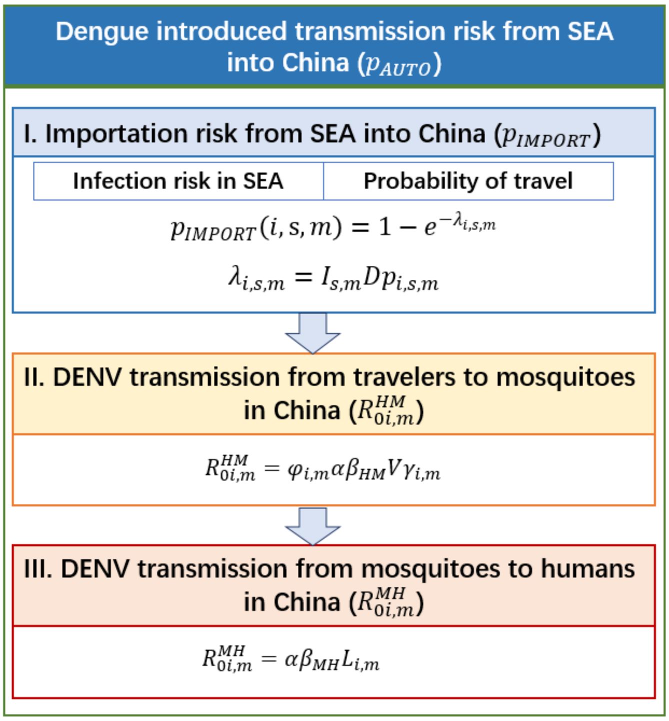

Parameters	
$I_{s,m}$	Incidence in origin *s* and month *m during the period of stay (C) in SEA*
D	Average duration of infection in humans
$p_{i,s,m}$	Probability of Chinese travelling to SEA and returning China or of SEA residents travelling to China
$\varphi_{i,m}$	Mosquitoes per person in location *i* and month *m*
α	Daily biting rate
β_{HM}	Human-to-mosquito transmissibility
β_{MH}	Mosquito-to-human transmissibility
V	Human infectious time
$\gamma_{i,m}$	Proportion of mosquitoes surviving the extrinsic incubation period in *i* and *m*
$L_{i,m}$	Period of an infectious mosquito surviving in *i* and *m*

Figure G. Model structure and parameters.

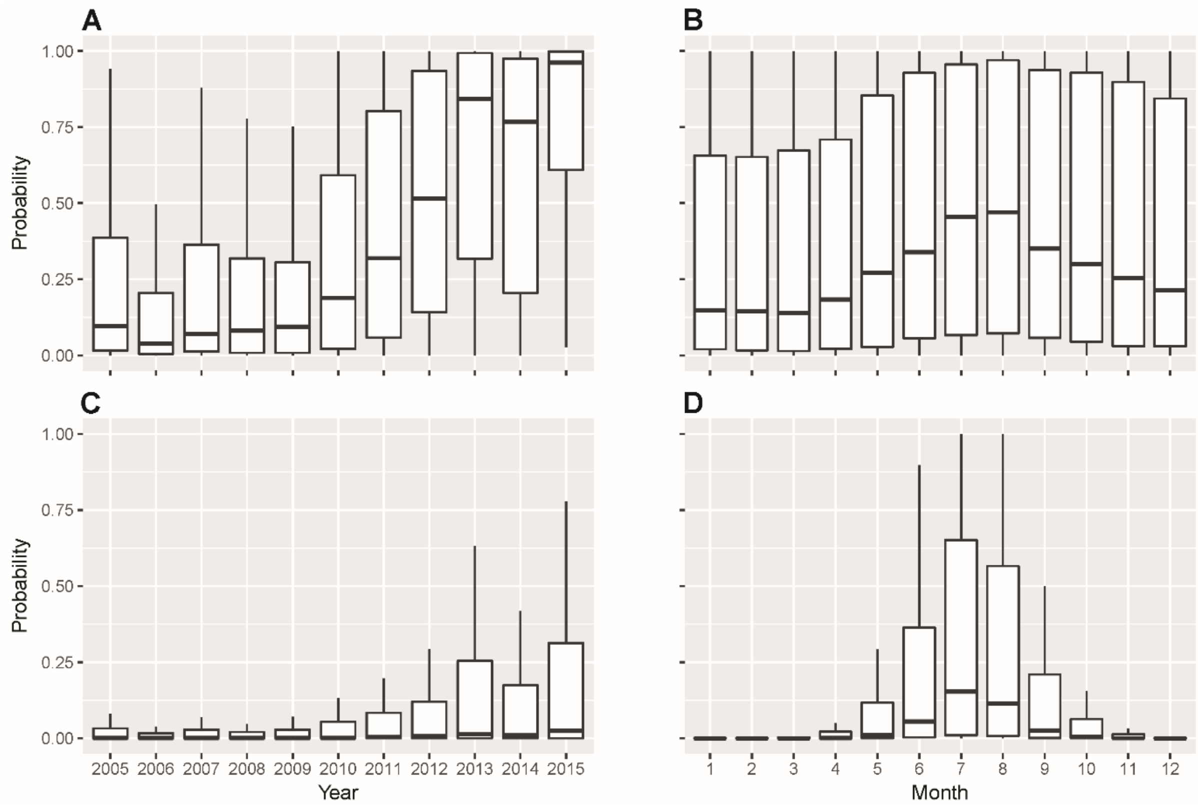

Figure H. Dengue importation and introduced transmission risks by Chinese travellers from South-East Asia into provinces of mainland China, 2005-2015. (A) Importation risk by year. (B) Importation risk by month. (C) Introduced transmission risk by year. (D) Introduced transmission risk by month. The probabilistic risk presented here is the likelihood of occurrence of at least one case, ranging from 0 (the lowest probability) to 1 (the highest) by 10,000 simulations. Nine countries (Cambodia, Laos, Malaysia, Maldives, Philippines, Singapore, Sri Lanka, Thailand, Vietnam) in SEA with available data of monthly DENV incidence were included here.

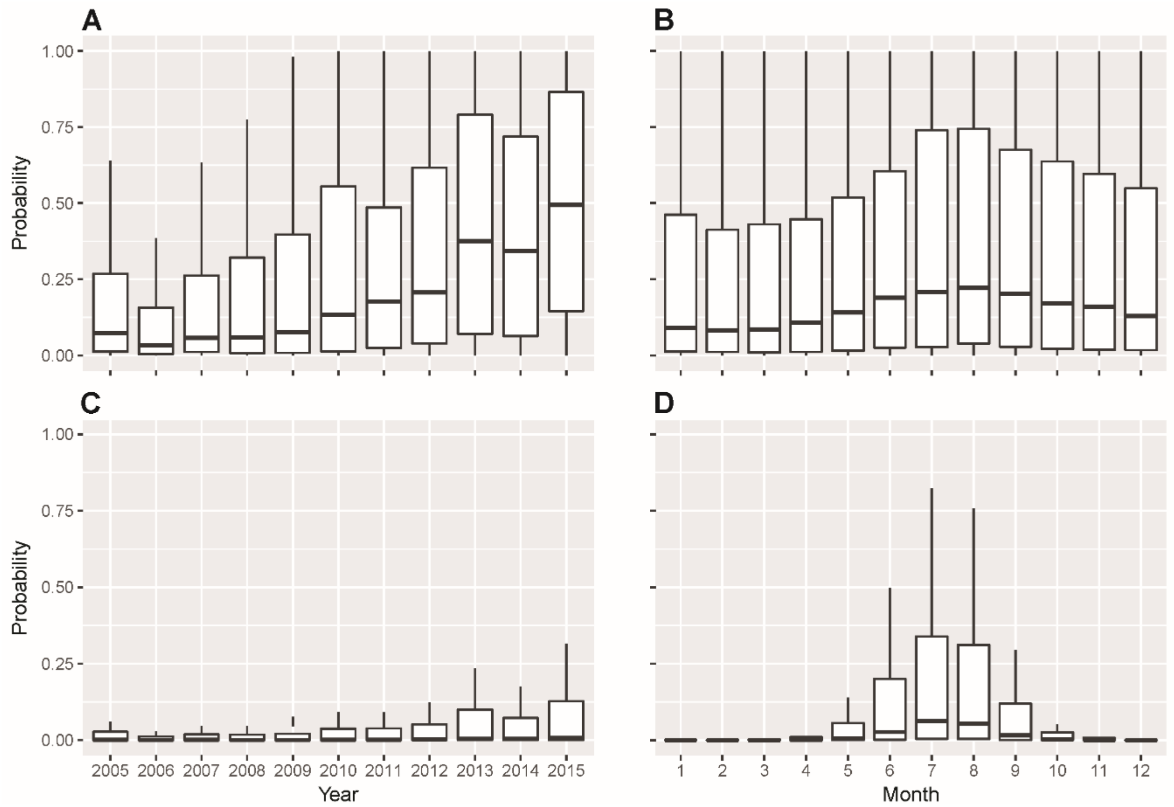

Figure I. Dengue importation and introduced transmission risks by residents from South-East Asia into provinces of mainland China, 2005-2015. (A) Importation risk by year. (B) Importation risk by month. (C) Introduced transmission risk by year. (D) Introduced transmission risk by month. The probabilistic risk presented here is the likelihood of occurrence of at least one case, ranging from 0 (the lowest probability) to 1 (the highest) by 10,000 simulations. Nine countries (Cambodia, Laos, Malaysia, Maldives, Philippines, Singapore, Sri Lanka, Thailand, Vietnam) in SEA with available data of monthly DENV incidence were included here.

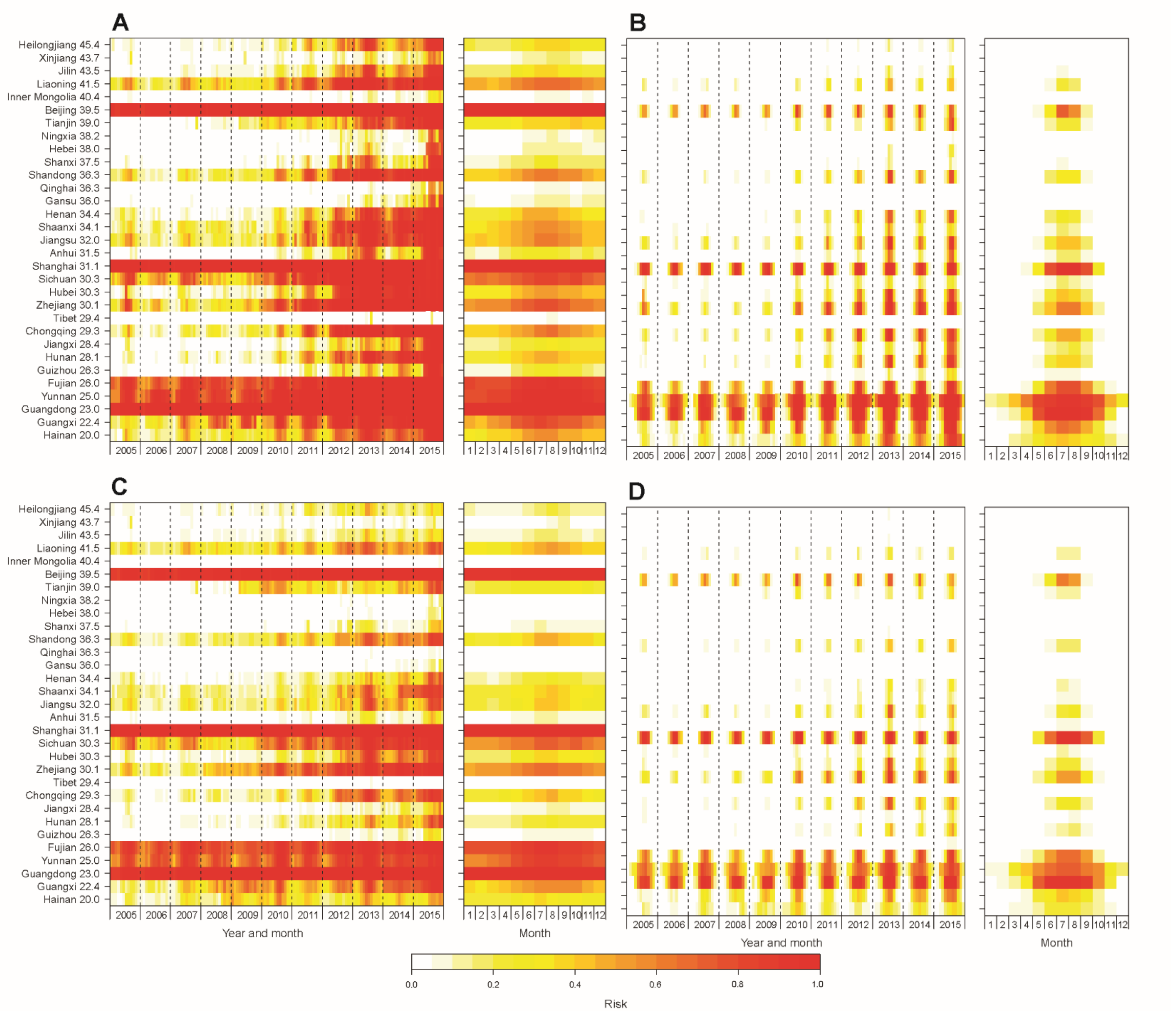

Figure J. Heatmaps of dengue importation and introduced transmission risk from nine SEA countries into provinces of China in 2005-2015, sorted by the latitude. (A) Risk of importation via Chinese. (B) Risk of importation via SEA residents. (C) Risk of introduced transmission via Chinese. (D) Risk of introduced transmission via SEA residents. The seasonal distribution of risk in each graph was plotted as the mean value of risk in each month of the year from 2005 to 2015.

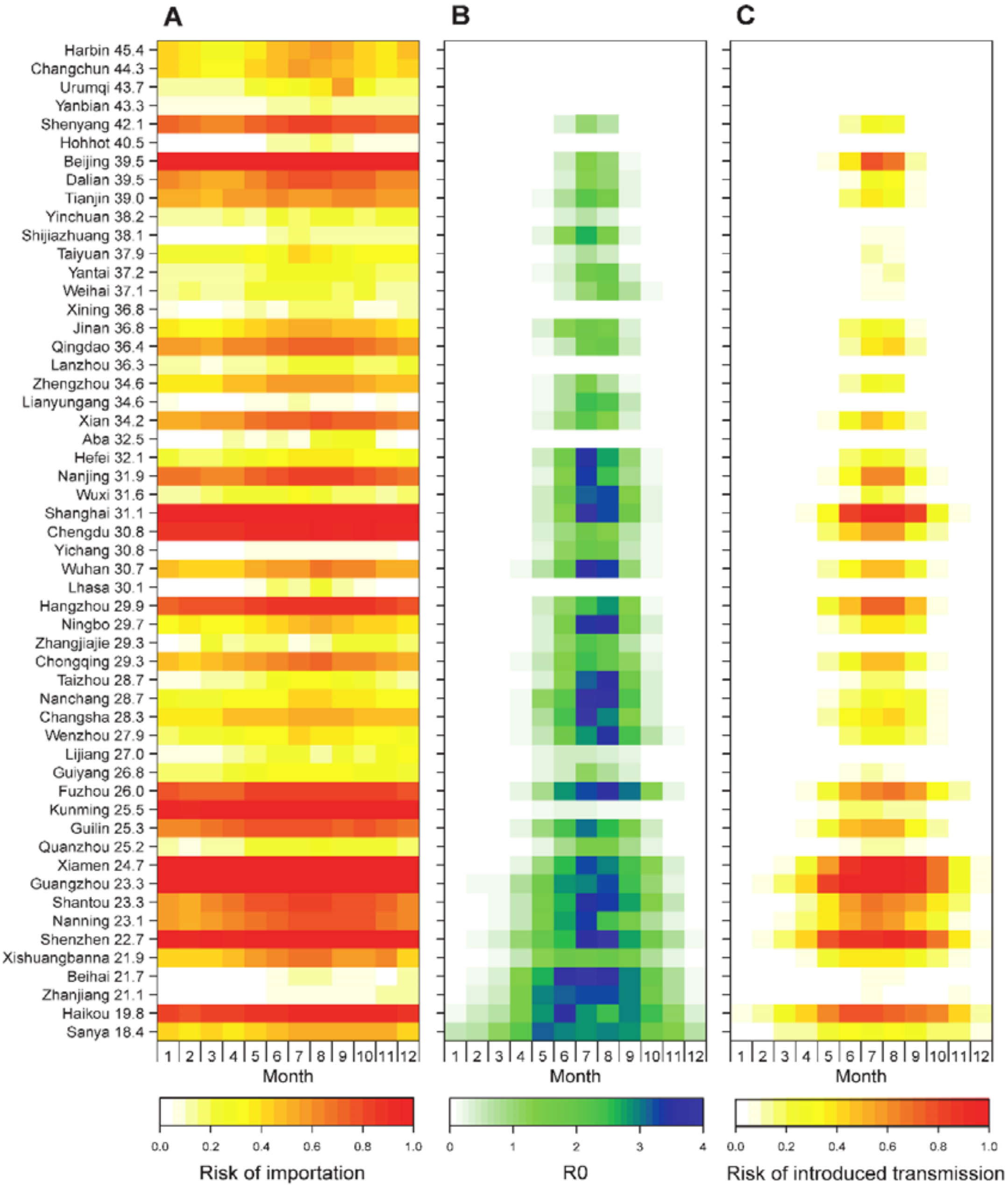

Figure K. Average monthly risks of dengue importation and introduced transmission from nine SEA countries into cities of China in 2005-2015, sorted by the latitude of cities. Only cities with the average importation risk greater than 0.05 from 2005 to 2015 were presented here. (A) Risk of importation. (B) Basic reproduction number (R0). (C) Risk of introduced transmission. $R_0$is defined as the product of R_0^{HM} and R_0^{MH} in a location *i* and month *m*.

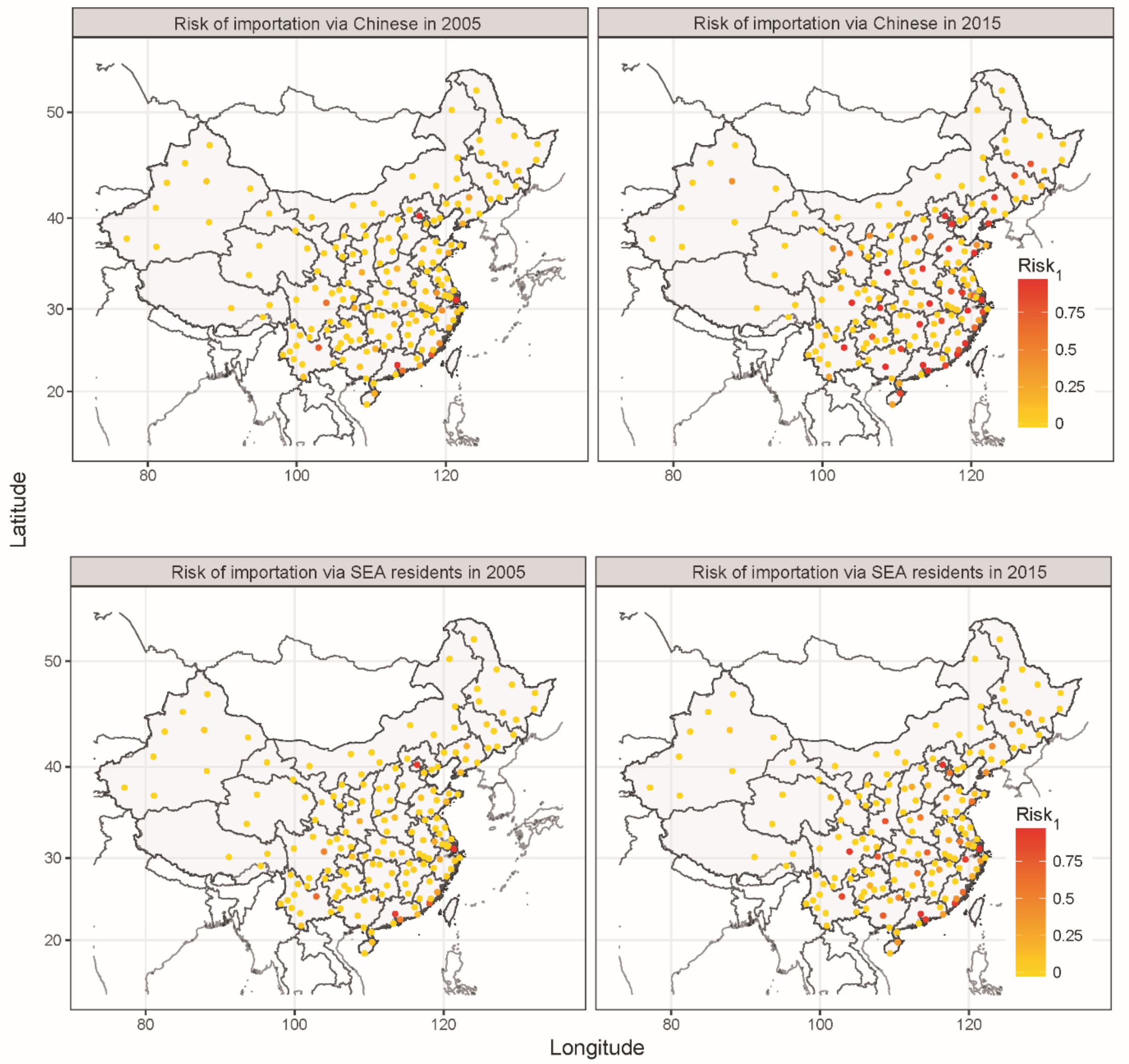

Figure L. Geographic risks of dengue importation by Chinese travellers and residents from South-East Asia into cities of mainland China in 2005 and 2015. The probabilistic risk (0-1) of importation DENV transmission from the nine SEA countries (Cambodia, Laos, Malaysia, Maldives, Philippines, Singapore, Sri Lanka, Thailand, Vietnam) with available monthly dengue incidence data.

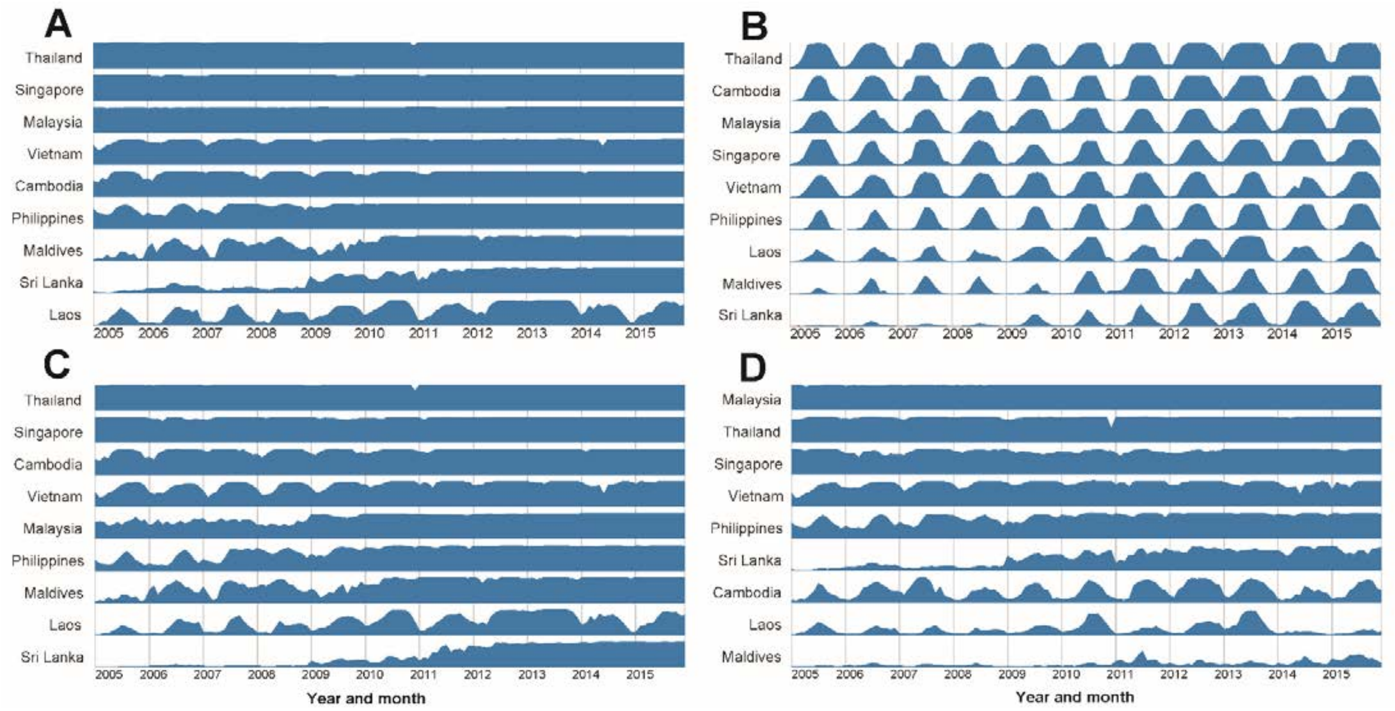

Figure M. Dengue importation and introduced transmission risks into China by origin country, 2005-2015. **(A)** Importation risk. **(B)** Risk of importation leading to autochthonous transmission in China. **(C)** Importation risk of Chinese travelers. **(D)** Importation risk of SEA resident travelling into China. Nine countries with available data of monthly DENV incidence were included and sorted by the average monthly risk in each graph.

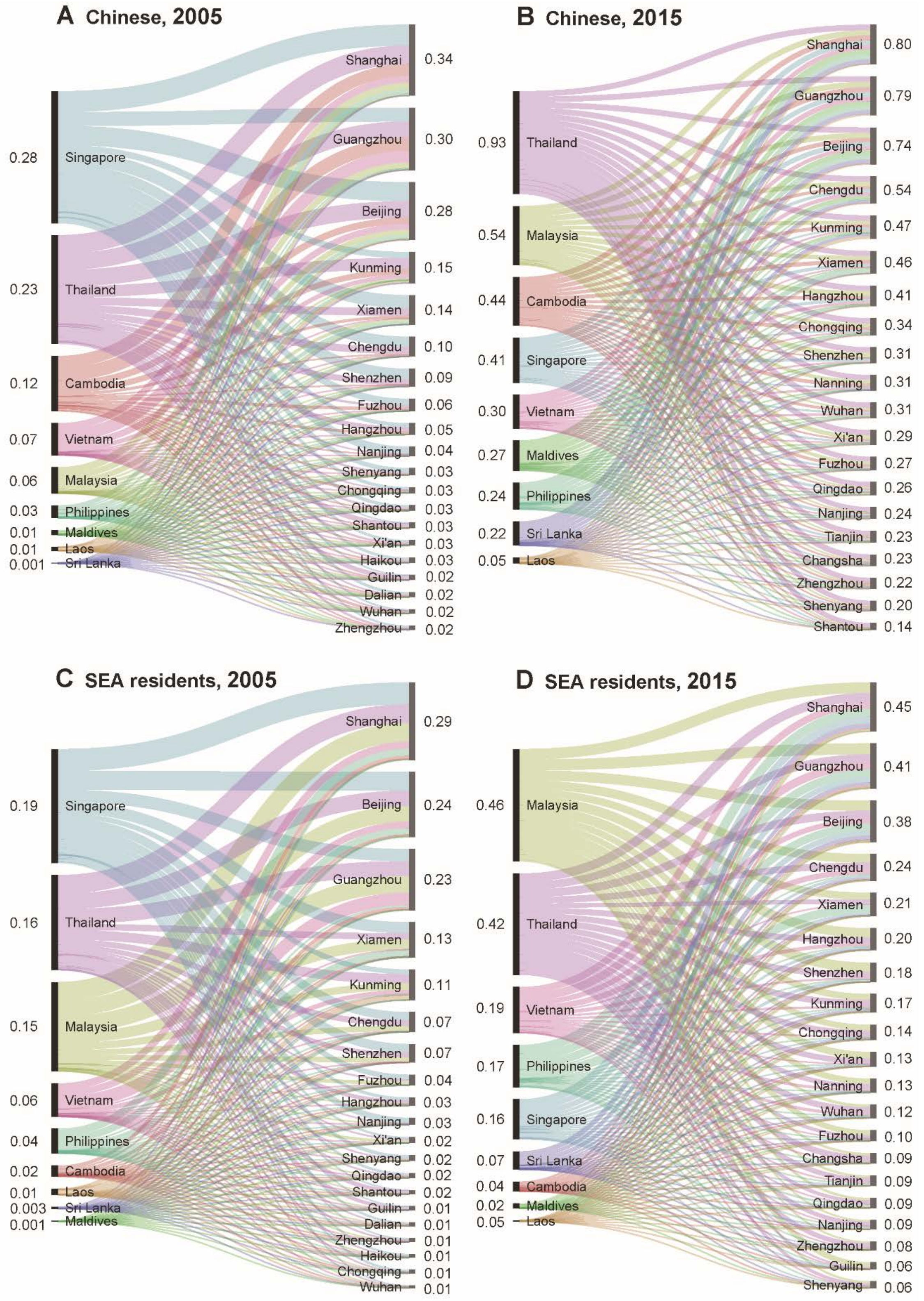

Figure N. Routes of dengue importation by nationality from South-East Asia into top 20 high-risk cities of China in 2005 and 2015. (A) Importation risk by Chinese travellers in 2005. (B) Importation risk by Chinese travellers in 2015. (C)

Importation risk by SEA residents travelling into China in 2005. (D) Importation risk by SEA residents travelling into China in 2015. The numbers in the figures are the average risk of all routes from/into each origin/destination. The thickness of line for each route is scaled to the risk from the lowest value (thinnest) to the highest value (thickest) within each panel.

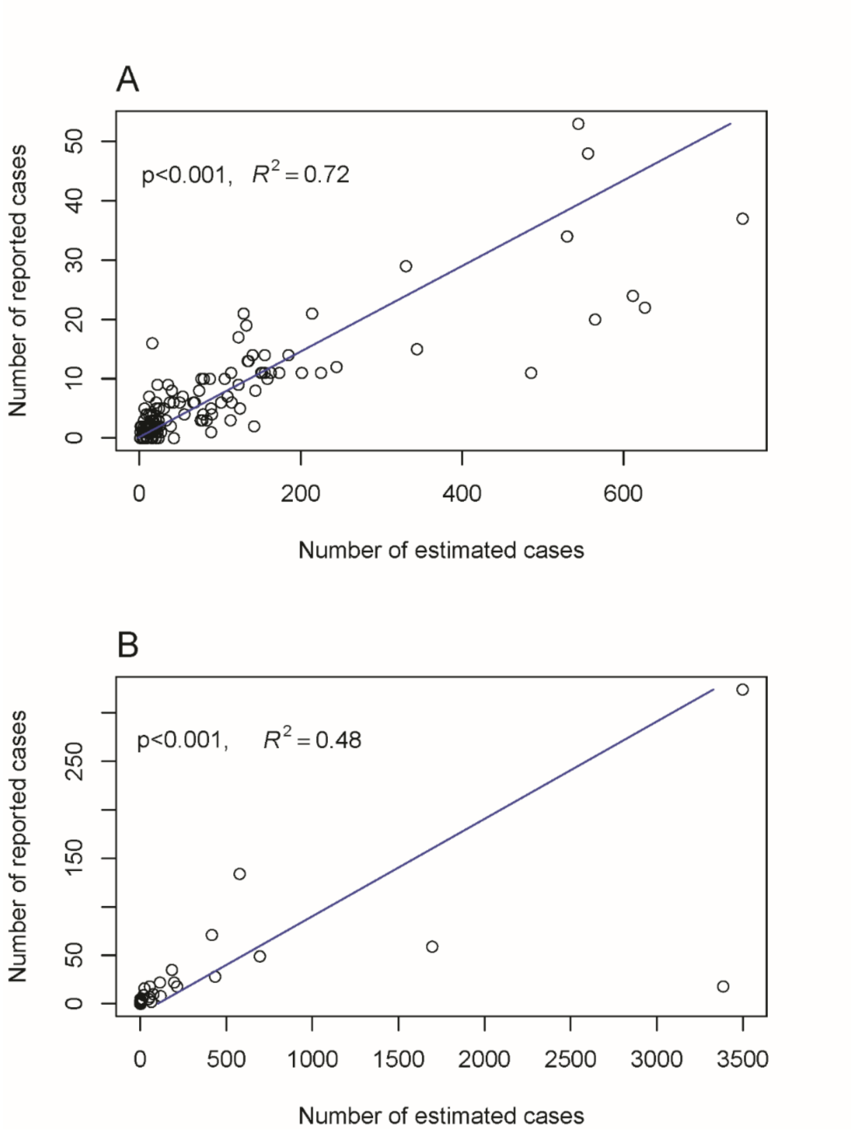

Figure O. The scatterplots of estimated dengue infections in airline travellers vs the number of imported cases reported in China, 2005-2015. (A) The scatterplot by year and month. (B) The scatterplot by province of China.

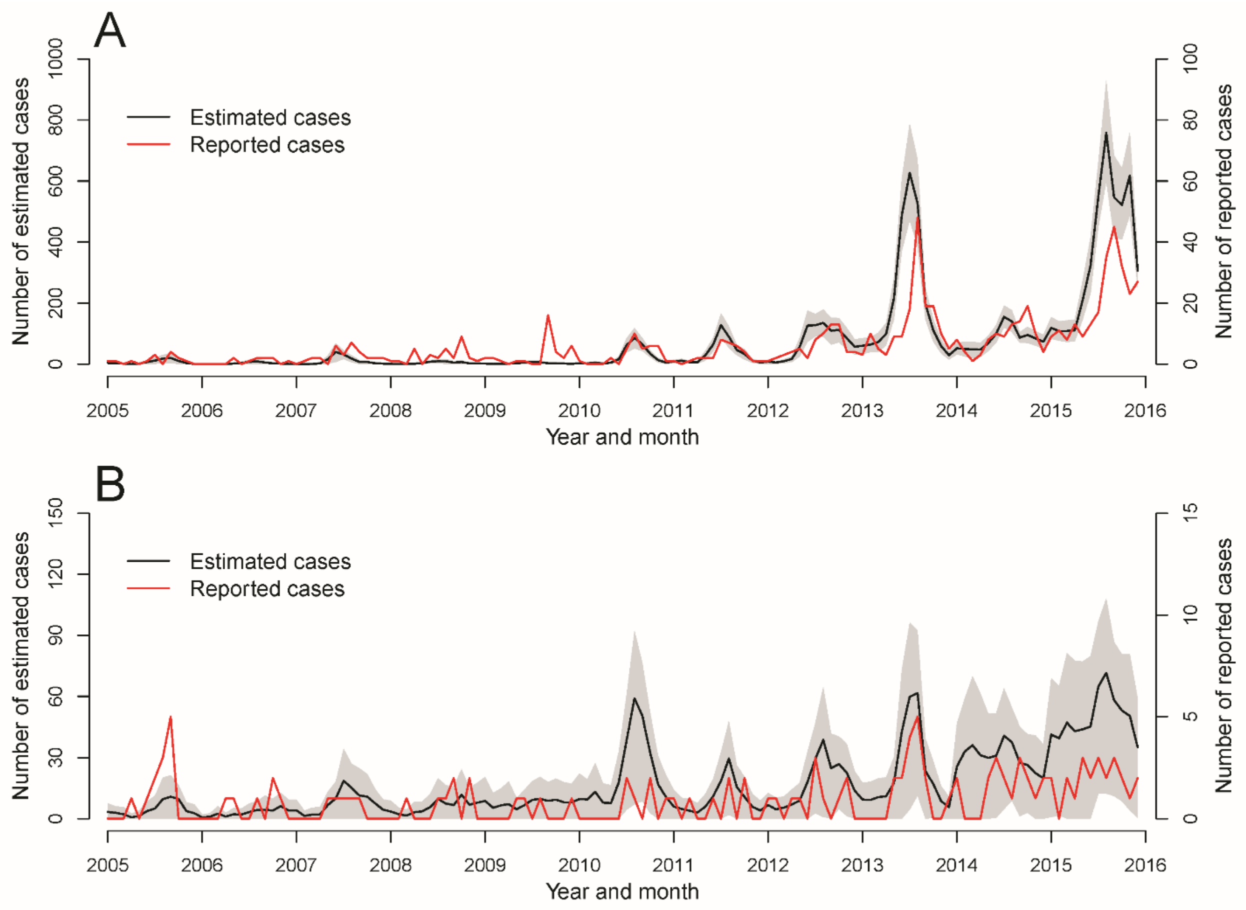

Figure P. The time series of estimated dengue infections in travellers and the number of imported cases reported in China. (A) Chinese. (B) SEA residents. The 95%CI of estimated infections are also provided.

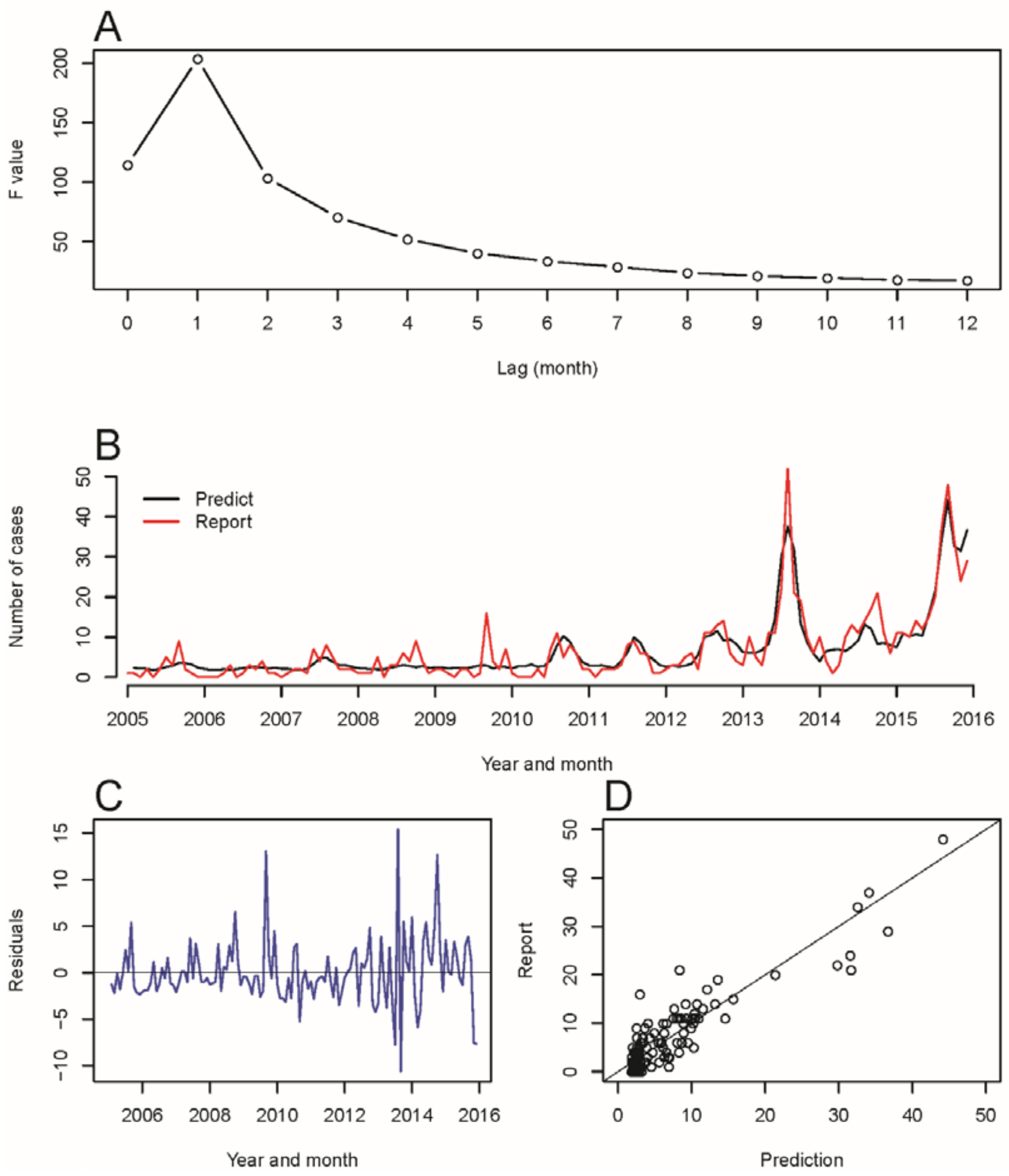

Figure Q. The performance of estimated dengue infections for predicting the time-series of cases reported in China. (A) F values of prediction using Granger causality test with a time lag from 0 to 12 months. (B) The performance of estimated infections to predict cases reported by surveillance using Granger causality test with one-month lag. (C) The residuals of prediction in (B). (D) The scatterplot of predicted cases vs reported cases by year and month.

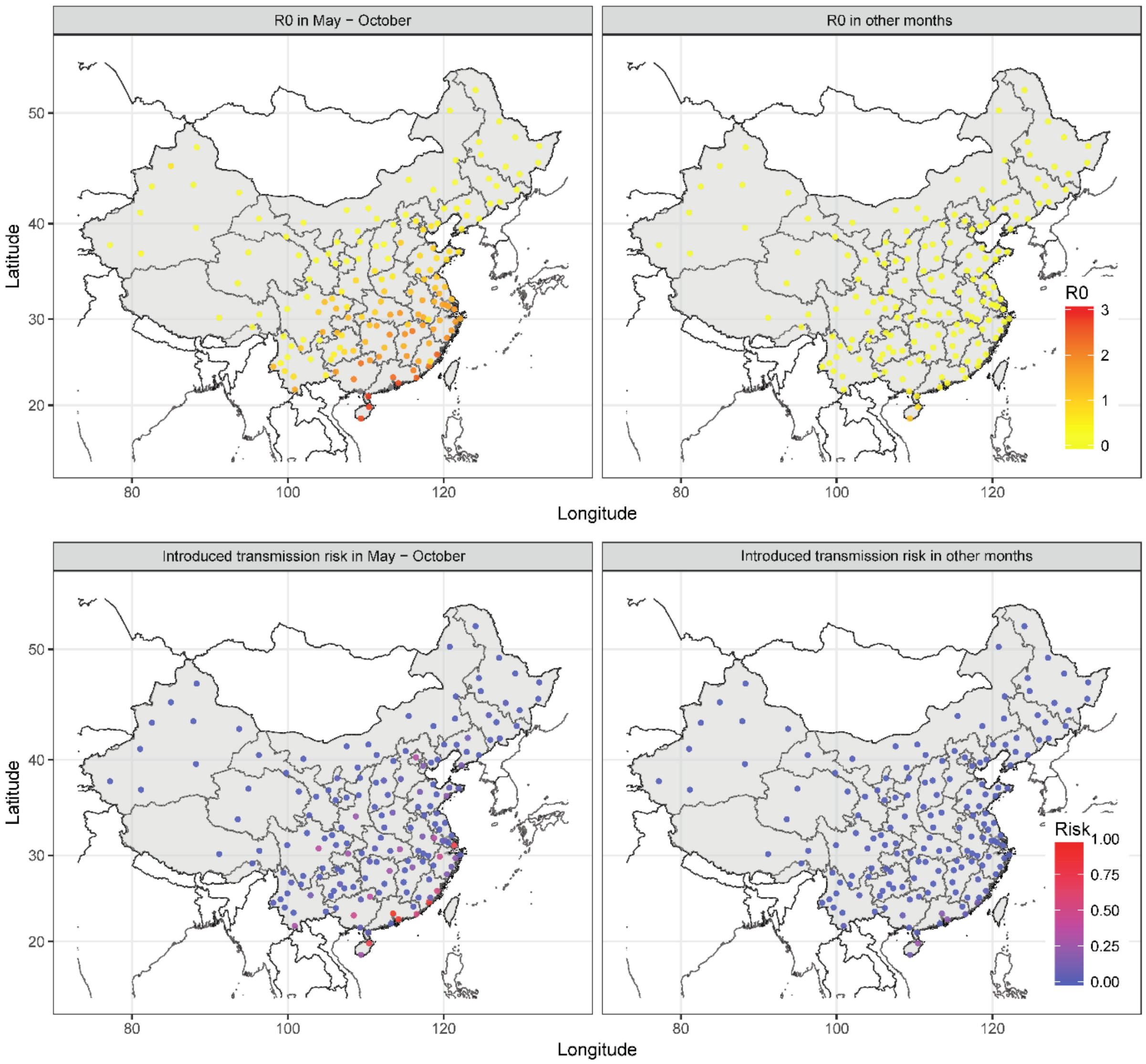

Figure R. Average basic reproduction number (R0) and DENV introduced transmission risk from South-East Asia into cities of China by season, 2005-2015. Nine countries (Cambodia, Laos, Malaysia, Maldives, Philippines, Singapore, Sri Lanka, Thailand, Vietnam) with available data of monthly DENV incidence were included as origins. $R_0$is defined as the product of R_0^{HM} and R_0^{MH} in a location *i* and month *m*.

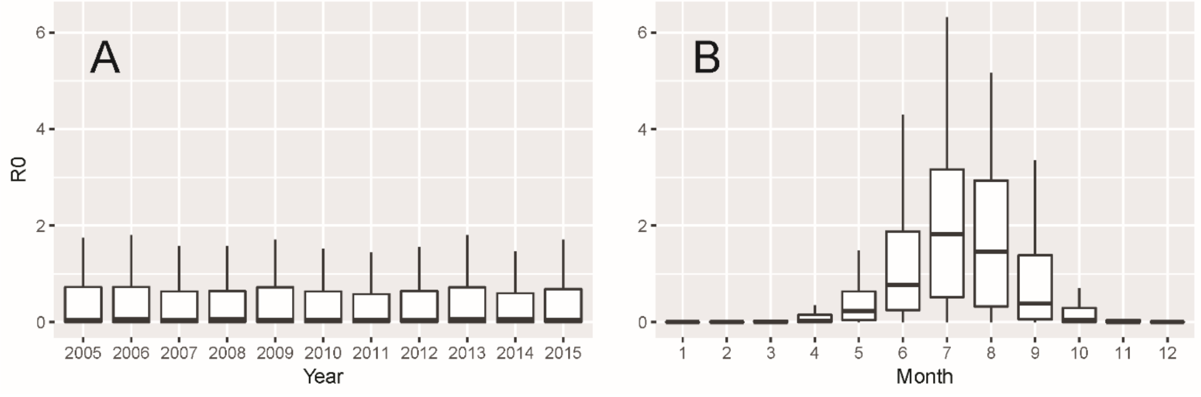

Figure S. The basic reproduction number (R0) of dengue transmission in China, 2005-2015. **(A)** R0 by year. **(B)** R0 by month. The R0 was estimated at provincial level by year and month. $R_0$is defined as the product of R_0^{HM} and R_0^{MH} in a location *i* and month *m*.

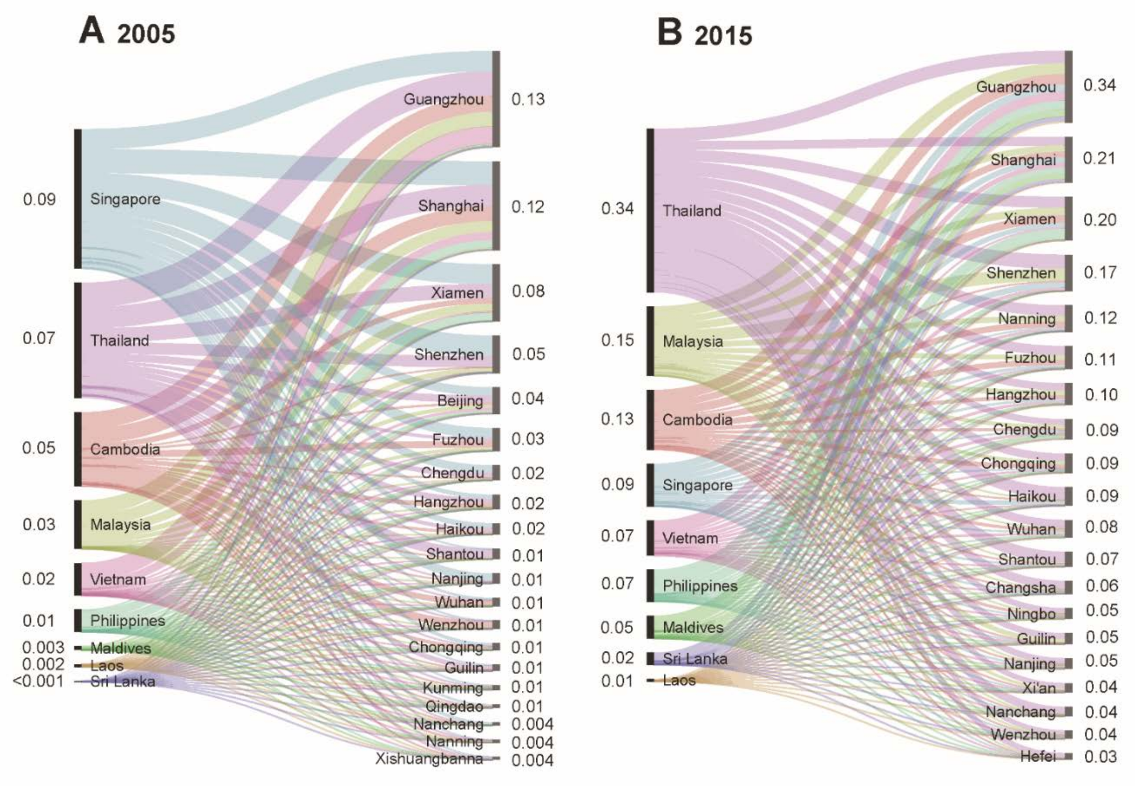

Figure T1. Origin-destination routes of dengue introduced transmission by imported infections from SEA into top 20 high-risk cities of China in 2005 and 2015. (A) Introduced transmission risk in 2005. (B) Introduced transmission risk in 2015. The numbers in the figures are the average risk of all routes from/into each origin/destination. The thickness of line for each route is scaled to the risk from the lowest value (thinnest) to the highest value (thickest) within each figure.

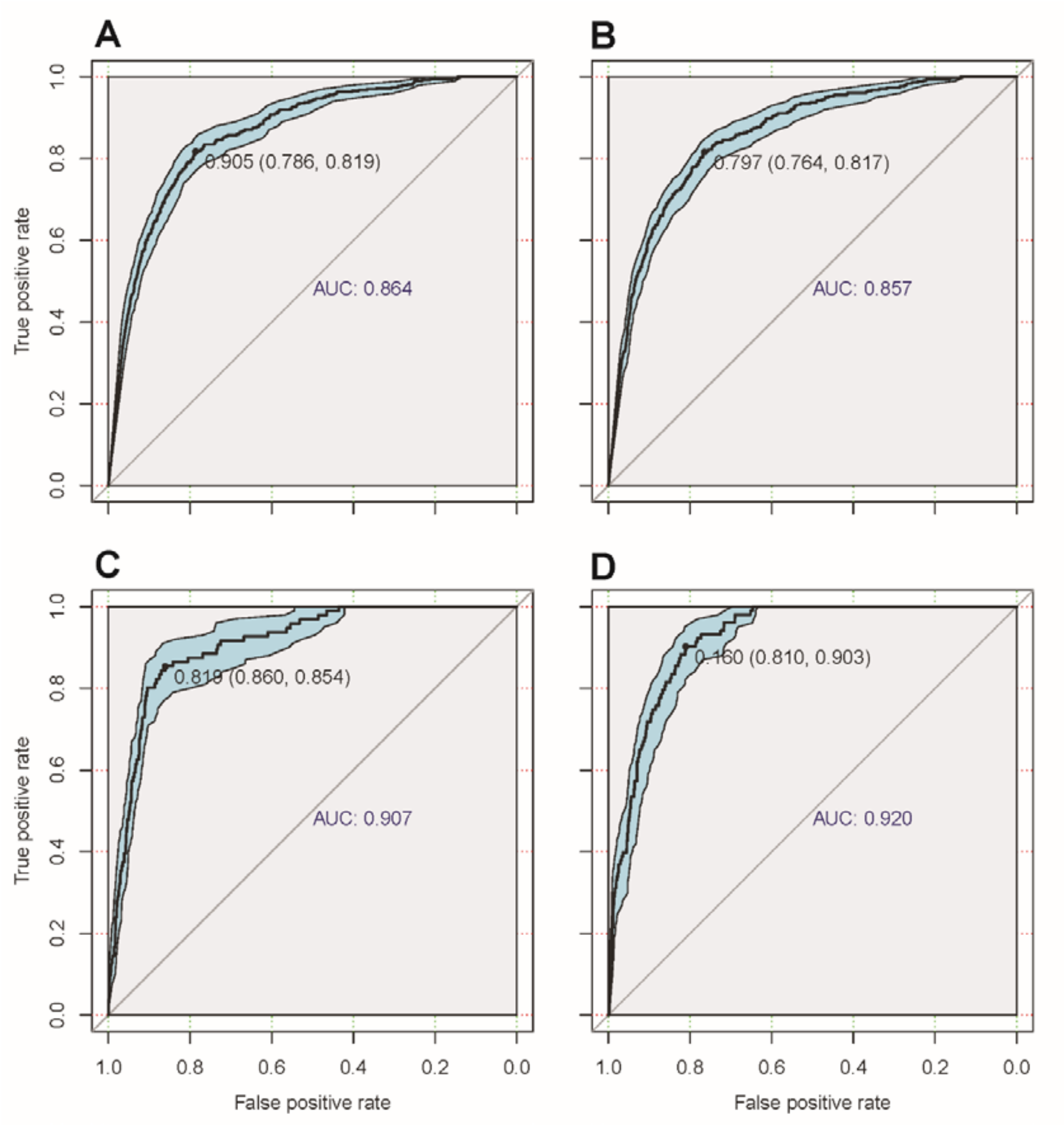

Figure U. ROC curve and AUC of estimated risks by comparing with the occurrence of imported and locally transmitted dengue at provincial level in China. (A) Importation risk by all airline travellers from nine SEA countries. (B) Importation risk via Chinese travellers. (C) Importation risk via SEA residents travelling into China. (D) Introduced transmission risk by all airline travellers. The 95% CI of ROC curve were generated by 1000 stratified bootstrap replicates.

References

1. Johansson MA, Arana-Vizcarrondo N, Biggerstaff BJ, Staples JE, Gallagher N, Marano N. On the treatment of airline travelers in mathematical models. PLoS One. 2011;6(7):e22151. http://dx.doi.org/10.1371/journal.pone.0022151

2. Carrasco LR, Lee LK, Lee VJ, Ooi EE, Shepard DS, Thein TL, et al. Economic impact of dengue illness and the cost-effectiveness of future vaccination programs in Singapore. PLoS Negl Trop Dis. 2011;5(12):e1426. http://dx.doi.org/10.1371/journal.pntd.0001426

3. Shepard DS, Undurraga EA, Halasa YA. Economic and disease burden of dengue in Southeast Asia. PLoS Negl Trop Dis. 2013;7(2):e2055. http://dx.doi.org/10.1371/journal.pntd.0002055

4. Undurraga EA, Halasa YA, Shepard DS. Use of expansion factors to estimate the burden of dengue in Southeast Asia: a systematic analysis. PLoS Negl Trop Dis. 2013;7(2):e2056. http://dx.doi.org/10.1371/journal.pntd.0002056

5. Grange L, Simon-Loriere E, Sakuntabhai A, Gresh L, Paul R, Harris E. Epidemiological risk factors associated with high global frequency of inapparent dengue virus infections. Front Immunol. 2014;5:280. http://dx.doi.org/10.3389/fimmu.2014.00280

6. Clapham HE, Cummings DAT, Johansson MA. Immune status alters the probability of apparent illness due to dengue virus infection: Evidence from a pooled analysis across multiple cohort and cluster studies. PLoS Negl Trop Dis. 2017;11(9):e0005926. http://dx.doi.org/10.1371/journal.pntd.0005926

7. van Panhuis WG, Choisy M, Xiong X, Chok NS, Akarasewi P, Iamsirithaworn S, et al. Region-wide synchrony and traveling waves of dengue across eight countries in Southeast Asia. Proc Natl Acad Sci U S A. 2015;112(42):13069-74. http://dx.doi.org/10.1073/pnas.1501375112

8. Cazelles B, Chavez M, Magny GC, Guegan JF, Hales S. Time-dependent spectral analysis of epidemiological time-series with wavelets. J R Soc Interface. 2007;4(15):625-36. http://dx.doi.org/10.1098/rsif.2007.0212

9. Torrence C, Compo GP. A Practical Guide to Wavelet Analysis. Bulletin of the American Meteorological Society. 1998;79(1):61-78. http://dx.doi.org/10.1175/1520-0477(1998)079<0061:apgtwa>2.0.co;2

10. Grinsted A, Moore JC, Jevrejeva S. Application of the cross wavelet transform and wavelet coherence to geophysical time series. Nonlin Processes Geophys. 2004;11(5/6):561-6. http://dx.doi.org/10.5194/npg-11-561-2004

11. Johansson MA, Arana-Vizcarrondo N, Biggerstaff BJ, Gallagher N, Marano N, Staples JE. Assessing the risk of international spread of yellow fever virus: a mathematical analysis of an urban outbreak in Asuncion, 2008. Am J Trop Med Hyg. 2012;86(2):349-58. http://dx.doi.org/10.4269/ajtmh.2012.11-0432

12. Johansson MA, Powers AM, Pesik N, Cohen NJ, Staples JE. Nowcasting the spread of chikungunya virus in the Americas. PLoS One. 2014;9(8):e104915. http://dx.doi.org/10.1371/journal.pone.0104915

13. Guzman MG, Harris E. Dengue. Lancet. 2015;385(9966):453-65. http://dx.doi.org/10.1016/S0140-6736(14)60572-9

14. Brady OJ, Golding N, Pigott DM, Kraemer MU, Messina JP, Reiner RC, Jr., et al. Global temperature constraints on Aedes aegypti and Ae. albopictus persistence and competence for dengue virus transmission. Parasit Vectors. 2014;7:338. http://dx.doi.org/10.1186/1756-3305-7-338

15. Stanaway JD, Shepard DS, Undurraga EA, Halasa YA, Coffeng LE, Brady OJ, et al. The global burden of dengue: an analysis from the Global Burden of Disease Study 2013. Lancet Infect Dis. 2016;16(6):712-23. http://dx.doi.org/10.1016/S1473-3099(16)00026-8

16. Lai S, Huang Z, Zhou H, Anders KL, Perkins TA, Yin W, et al. The changing epidemiology of dengue in China, 1990-2014: a descriptive analysis of 25 years of nationwide surveillance data. BMC Med. 2015;13:100. http://dx.doi.org/10.1186/s12916-015-0336-1

17. Scott TW, Amerasinghe PH, Morrison AC, Lorenz LH, Clark GG, Strickman D, et al. Longitudinal studies of Aedes aegypti (Diptera: Culicidae) in Thailand and Puerto Rico: blood feeding frequency. J Med Entomol. 2000;37(1):89-101.

18. Chan M, Johansson MA. The incubation periods of Dengue viruses. PLoS One. 2012;7(11):e50972. http://dx.doi.org/10.1371/journal.pone.0050972

19. Brady OJ, Johansson MA, Guerra CA, Bhatt S, Golding N, Pigott DM, et al. Modelling adult Aedes aegypti and Aedes albopictus survival at different temperatures in laboratory and field settings. Parasit Vectors. 2013;6:351. http://dx.doi.org/10.1186/1756-3305-6-351
